# Long-term neurocognitive outcomes of SARS-CoV-2 infection in a hamster model

**DOI:** 10.3389/fmicb.2025.1646616

**Published:** 2025-08-14

**Authors:** Carla Ruiz-Casas, Ferran Tarrés-Freixas, Núria Roca, Mònica Pérez, Guillermo Cantero, Laura Martín, Alex Olvera, Marta Ruiz-Riol, Christian Brander, Carla Usai, Júlia Vergara-Alert, Joaquim Segalés

**Affiliations:** ^1^Unitat Mixta d’Investigació IRTA-UAB en Sanitat Animal, Centre de Recerca en Sanitat Animal (CReSA), Campus de la Universitat Autònoma de Barcelona (UAB), Bellaterra, Spain; ^2^IRTA Programa de Sanitat Animal, Centre de Recerca en Sanitat Animal (CReSA), Campus de la UAB, Bellaterra, Spain; ^3^Biosciences Department, Faculty of Sciences and Technology, University of Vic - Central University of Catalonia, Barcelona, Spain; ^4^Irsi-Caixa, Hospital Germans Trias i Pujol, Badalona, Spain; ^5^CIBERINFEC, Instituto de Salud Carlos III, Madrid, Spain; ^6^ICREA, Passeig Lluís Companys 23, Barcelona, Spain; ^7^Department de Sanitat i Anatomia Animals, Facultat de Veterinària, Campus de la UAB, Bellaterra, Spain

**Keywords:** post-COVID-19 condition, post-acute sequelae of COVID-19, golden Syrian hamster model, SARS-CoV-2 persistence, long-term neurological sequelae, anxiety-related behavioral tests

## Abstract

**Introduction:**

Post-COVID-19 condition (PCC) is a long-lasting, multisystemic syndrome affecting approximately 30% of individuals after acute COVID-19, with neurological symptoms among the most prevalent and debilitating. Despite its substantial global health impact, the biological mechanisms underlying PCC remain poorly understood, underscoring the need for validated animal models.

**Methods:**

To address this, we conducted a longitudinal study using the golden Syrian hamster model, integrating virological, immunological, histopathological, and behavioral analyses from the acute phase to 60 days post-inoculation.

**Results:**

Our results showed persistent viral RNA, prolonged immune dysregulation, and behavioral changes that mirrored key features of human PCC.

**Discussion:**

Although fully reproducing PCC in animal models is inherently challenging due to its complex and heterogeneous presentation in humans, the use of complementary models with distinct phenotypes is essential for elucidating its pathophysiology. These results aim to contribute valuable insights into the biological basis of PCC and support the development of targeted preventative and therapeutic strategies.

## Introduction

As of June 2025, severe acute respiratory syndrome coronavirus 2 (SARS-CoV-2) has caused over 777 million confirmed Coronavirus Disease 2019 (COVID-19) cases worldwide (WHO COVID-19 dashboard, n.d.). While most patients recover within a few weeks, approximately 30% experience persistent multisystemic symptoms, a condition referred to as Long-COVID, post-acute sequelae of COVID-19, or post-COVID-19 condition (PCC) (Fernandez-de-las-Peñas et al., 2024).

PCC typically occurs 3 months after SARS-CoV-2 infection and persists for at least 2 months, with symptoms that may be new or persist from the initial illness, often fluctuating or relapsing over time. It can affect people of all ages, with a higher prevalence in women, and can occur regardless of the initial disease severity (Soriano et al., 2022). PCC encompasses a broad spectrum of respiratory and extra-respiratory symptoms, including hematological, immune, cardiovascular, renal, gastrointestinal and hepatobiliary, endocrine, neurological, ophthalmological, and dermatological disorders (Usai et al., 2023). Among these, neurological manifestations (neuro-PCC)—such as fatigue, cognitive dysfunction, headaches, anosmia, sleep disturbances, depression, and anxiety—are particularly common and debilitating (Davis et al., 2021; Taquet et al., 2022; Mina et al., 2023).

Despite extensive clinical documentation, the underlying pathogenesis of neuro-PCC remains poorly understood (Greenhalgh et al., 2024). Proposed mechanisms include the persistence of SARS-CoV-2 or its components in tissues, lasting immune dysregulation, microbiota disruption, autoimmunity, clotting and endothelial dysfunction, and disrupted neurological signaling involving the brainstem and vagus nerve (Peluso and Deeks, 2024). These mechanisms may interact in complex ways, varying in combinations, frequencies, and timelines across individuals (Peluso and Deeks, 2024). Given the complexity of these interrelated processes, the potential existence of robust animal models would be key to advance our understanding of PCC pathophysiology. Animal models have been instrumental in uncovering the pathogenesis of acute COVID-19. During the early stages of the pandemic, naturally susceptible species, including non-human primates, golden Syrian hamsters (GSH), ferrets, and transgenic mice expressing human angiotensin-converting enzyme 2 (hACE2), the cellular receptor for SARS-CoV-2, were widely used to evaluate prophylactic and therapeutic strategies (Muñoz-Fontela et al., 2020). Among these, the GSH has emerged as particularly promising for investigating PCC, as it exhibits a mild-to-moderate, human-like acute illness without mortality, enabling long-term post-infection monitoring (Usai et al., 2023; Chen et al., 2024). Recent studies in GSH have linked SARS-CoV-2 infection to neurobiological alterations, including blood–brain barrier disruption, microgliosis, tau and alpha-synuclein accumulation in cortical neurons, alterations in parvalbumin and calretinin interneurons, reduced hippocampal neurogenesis, and elevated proinflammatory cytokines and chemokines (e.g., IL-6, IL-1β, CXCL10). Additionally, transcriptional dysregulation of key genes has been reported in the cerebrum (e.g., *Cd3e, C3, Mhc II, Cd68, Il18, Cxcl10, Il1b, Tnfa*), olfactory bulb (e.g., *Cd3e, Mhc II, Cd68, Cxcl10*), and thalamus (e.g., *Nrp1, App, Camk2a*) (Frere et al., 2022; Käufer et al., 2022; Soung et al., 2022; Roczkowsky et al., 2023; Schreiber et al., 2024). Notably, some of these changes have been linked to cognitive impairments and anxiety-like behaviors in GSH, as evidenced by behavioral tests (Frere et al., 2022).

Here, we investigated whether SARS-CoV-2 infection in GSH induces long-term neurological alterations consistent with PCC. To our knowledge, this study represents the longest follow-up of neurocognitive outcomes in this model, extending to 60 days post-inoculation (dpi). By integrating virological, immunological, histopathological, and behavioral analyses, we aimed to identify central and peripheral neuropathological signatures of PCC. Anticipating individual variability, mirroring the heterogeneity observed in human PCC, we prioritized individual-level over group-based analyses. These findings are intended to provide novel insights to support the development of diagnostic and therapeutic strategies for neuro-PCC.

## Materials and methods

### Ethics statements

The experimental study protocol was approved by the Institutional Animal Welfare Committee of the *Institut de Recerca i Tecnologia Agroalimentàries* (CEEA-IRTA, registration number CEEA 384-2023) and the Ethical Commission of Animal Experimentation of the Government of Catalonia (ethical approval n°: CEA-OH/12097/2). The study was conducted by certified staff, and all experiments involving SARS-CoV-2 were performed at the BSL-3 facilities of the Biocontainment Unit of IRTA-CReSA (Barcelona, Spain).

### Virus isolates and cells

The SARS-CoV-2 isolate used in these studies, identified as Cat02 (GISAID ID EPI_ISL-471472), was isolated from a nasopharyngeal aspirate of a laboratory-confirmed COVID-19 patient in Barcelona, Spain, in 2020. This B.1 lineage isolate has the following point mutations compared to the Wuhan strain: D614G in the Spike protein, K837N in NSP3, and P323L in NSP12.

Production of virus stock (passage number 3), isolation, titration, and virus neutralization assays were conducted using Vero E6 cells (ATCC® repository, CRL-1586™). The infectious virus titer was determined by calculating the 50% Tissue Culture Infectious Dose (TCID_50_) endpoint, and results were expressed as TCID_50_/mL (Reed and Muench, 1938).

The preparation of virus and cells was conducted as previously described (Brustolin et al., 2021), using Dulbecco’s modified Eagle Medium (DMEM; Lonza) supplemented with 5% fetal bovine serum (FBS; 35-079-CV, Corning^®^), 100 U/mL penicillin, 100 μg/mL streptomycin, and 2 mM glutamine (all from Gibco Life Technologies).

### Study design

**Figure 1:**
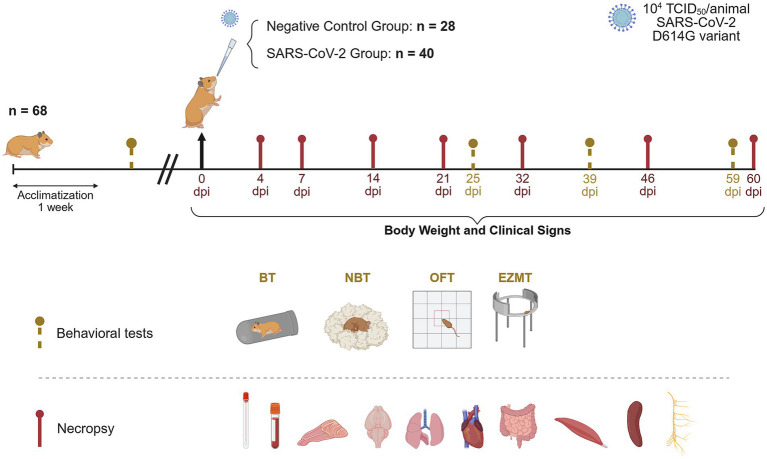
Experimental design for long-term monitoring of SARS-CoV-2 infection in golden Syrian hamsters. Forty golden Syrian hamsters (20 males and 20 females) were intranasally instilled with 10^4^ tissue culture infectious dose (TCID_50_) of the SARS-CoV-2 Cat02 isolate. Viral shedding in oropharyngeal swabs, clinical signs and body weight were monitored prior to inoculation and throughout the study. Behavioral tests, including the burrowing (BT), nest building (NBT), open field (OFT), and elevated zero maze (EZMT) tests were conducted before the challenge and at 25, 39, and 59 days post-inoculation (dpi) to assess potential SARS-CoV-2-associated behavioral changes. At necropsy, plasma, oropharyngeal swabs, nasal turbinate, brain, lung, heart, small intestine, muscle, spleen, and cervical tissue were collected for virological, immunological and/or histopathological analyses (created with https://BioRender.com).

The experimental design is depicted in Figure 1. A total of 68 GSH (Janvier Labs), aged 6-to-8 weeks, including 34 males and 34 females, were used in this study. Following a one-week acclimatization period, 40 hamsters (20 males and 20 females) were intranasally inoculated under isoflurane anesthesia with 100 μL of SARS-CoV-2 Cat02 isolate (10^4^ TCID_50_, 50 μL per nostril). An additional 28 hamsters (14 males, 14 females) were intranasally mock-inoculated with the same volume of phosphate-buffered saline (PBS) and served as negative controls.

Clinical signs and body weight were recorded prior to challenge, daily until 10 dpi, three times per week from 11 to 21 dpi, and weekly thereafter until 60 dpi. Oropharyngeal swabs (OS) were collected before challenge and weekly up to 60 dpi for quantitative analysis of SARS-CoV-2 viral shedding.

Neurocognitive function was assessed using four behavioral tests: nest-building test (NBT), burrowing test (BT), open field test (OFT), and elevated zero maze test (EZMT), conducted pre-inoculation and at 25, 39, and 59 dpi. The NBT and BT evaluated innate behaviors that are sensitive to brain lesions, motor deficits, and general welfare (Deacon, 2012), while the OFT and EZMT assessed locomotor activity and anxiety-related behaviors (Shepherd et al., 1994; Seibenhener and Wooten, 2015).

For necropsy and sample collection, four mock-inoculated and four SARS-CoV-2-inoculated hamsters (with equal numbers of males and females) were euthanized via intraperitoneal pentobarbital injection at 4, 7, 14, and 21 dpi. The same procedure was performed at 32, 46, and 60 dpi, using four controls and eight inoculated hamsters at each time point (with equal numbers of males and females).

### Sample collection, processing, and storage

For immunological and virological analyses, hamsters were sampled for blood, OS, and tissue samples, including the left nasal turbinate, left caudal hemibrain, left caudal lung, heart, small intestine, right hind limb muscle, and spleen.

Blood was collected into 2 mL cryotubes containing 200 μL 0.5 M EDTA, then centrifuged at 1,000 × g for 10 min (min) to separate plasma. Plasma was aliquoted into 1.5 mL cryotubes and stored at −80 °C.

OS and tissue samples (except for the spleen) were placed in 2 mL cryotubes containing 1,000 μL of DMEM (Lonza, Basel, Switzerland) supplemented with 100 U/mL penicillin, 100 μg/mL streptomycin, and 2 mM glutamine (all from Gibco Life Technologies, Madrid, Spain). OS were resuspended by vortexing for 30 s (s). A 3 mm tungsten bead (69997, QIAGEN, Hilden, Germany) was added to tissue samples except the spleen, which were homogenized at 25 Hz for 30 s using a TissueLyser II (QIAGEN, Hilden, Germany), then centrifuged at 2,000 × g for 2 min. Samples were stored at −80 °C until further analysis.

Hamster spleens were collected in RPMI 1640 (11875093, ThermoFisher Scientific), supplemented with 20% FBS (35-079-CV, Corning^®^), 1% glutamine, and 1% penicillin/streptomycin (all from Gibco Life Technologies). All steps were performed on ice. Splenocytes were isolated using a combination of mechanical and enzymatic digestion. Briefly, spleens were dissociated by gentle straining through a 40 μm cell strainer (352340, Corning^®^). Red blood cells were lysed using a red blood cell lysis buffer (17 mM TRIS, 0.14 M NH_4_Cl, adjusted to pH 7.4). Splenocytes were washed and resuspended in FBS (35-079-CV, Corning^®^) containing 10% dimethyl sulfoxide (DMSO; D2650, Sigma-Aldrich™) and quickly transferred to cryovials gradually frozen to −80 °C overnight. Samples were then transferred to liquid nitrogen (−196 °C) for long-term storage until further use.

For histopathological examination, the right nasal turbinate, right hemibrain, right lung, heart, small intestine, right posterior limb muscle, and cervical region containing the cervical vagus nerve (CVN) were collected in specific containers (3178–600-01, Biopsafe®, Axlab, Vedbæk, Denmark) and fixed by immersion in 10% buffered formalin for 7 days prior to processing.

### RNA-extraction and detection by RT-qPCR

Viral RNA was extracted using the IndiMag Pathogen Kit (Indical Bioscience) on a Biosprint 96 workstation (QIAGEN), following the manufacturer’s instructions. Detection of SARS-CoV-2 genomic RNA (gRNA) was performed by reverse transcription quantitative polymerase chain reaction (RT-qPCR) on OS, nasal turbinate, lung, brain, heart, intestine, and muscle samples, as previously described (Corman et al., 2020).

RT-qPCR was conducted using AgPath-ID™ One-Step RT-PCR Reagents (Applied Biosystems, Life Technologies), targeting a segment of the envelope protein gene (UpE; GenBank NC_004718, positions 26,141–26,253). The assay employed the forward primer (5′-ACAGGTACGTTAATAGTTAATAGCGT-3′ [400 nM]), reverse primer (5′-ATATTGCAGCAGTACGCACACA-3′ [400 nM]), and probe (5′-FAM-ACACTAGCCATCCTTA CTGCGCTTCG-TAMRA-3′ [200 nM]). Thermal cycling conditions included reverse transcription at 50 °C for 10 min, initial denaturation at 95 °C for 10 min, followed by 45 cycles of 94 °C for 15 s (s) and 58 °C for 30 s on a 7,500 Fast Real-Time PCR System (Applied Biosystems, Life Technologies).

For absolute quantification, a standard curve was generated using serial 10-fold dilutions of an E gene plasmid (10006896, 2019-nCoV_E_Positive Control, IDT), run in parallel with all PCR assays. Each sample was analyzed in duplicate, with Ct differences of less than one cycle considered acceptable.

### Viral titration in Vero E6 cells

Supernatants from lysed samples exhibiting Ct values lower than 36 were analyzed for infectious virus by titration in Vero E6 cells, as previously described (Brustolin et al., 2021). Briefly, each sample was serially diluted 10-fold in duplicate and transferred to a 96-well plate pre-seeded with Vero E6 cells. Following incubation at 37 °C with 5% CO_2_ for 5 days, plates were examined for cytopathic effect, and the infectious viral titer was determined by calculating the TCID_50_, according to the Reed-Muench method (Reed and Muench, 1938).

### Histopathological and immunohistochemical analyses

After fixation, the nasal turbinate, brain, lung, heart, intestine, muscle, and cervical region were routinely processed to obtain 4 μm-thick, formalin-fixed, paraffin-embedded sections for histopathology. Prior to standard processing, nasal turbinate and cervical region samples underwent decalcification in decalcifying solution (10-6553, Casa Álvarez) for 10 and 24 h (h), respectively. Cervical region samples were then sectioned into 2 mm-thick slices, placed in embedding cassettes, and subjected to a second decalcification cycle.

Hematoxylin and eosin (H&E) staining was performed on all tissue sections to assess inflammation resulting from SARS-CoV-2 infection using an optical microscope. Inflammation severity was scored with a semi-quantitative system (0–3), based on previously published criteria (Brustolin et al., 2021; Vidal et al., 2022): 0 = absent, 1 = mild, 2 = moderate, and 3 = severe.

Immunohistochemistry (IHC) was conducted on sections of nasal turbinate, lung, brain, heart, intestine, and muscle to detect SARS-CoV-2 nucleocapsid (N) protein using a rabbit monoclonal antibody (40143-R019, Sino Biological) at a 1:10000 dilution (Rockx et al., 2020). Viral antigen levels were assessed using a semi-quantitative scale (0–3), based on previously described criteria (Brustolin et al., 2021; Vidal et al., 2022): 0 = absent, 1 = low, 2 = moderate, and 3 = high.

H&E-stained sections from the cervical region of animals euthanized at 60 dpi were digitized using a slide scanner (Aperio, Leica Biosystems) and assessed blindly with Aperio ImageScope software (v12.4.6.5003, Leica Biosystems). The CVN was identified based on its anatomical location: dorsolateral to the carotid artery, medial to the jugular vein, and adjacent to the sympathetic trunk (Planitzer et al., 2017). At a standardized anatomical level, defined by the presence of thymic tissue, the diameter of the CVN was measured perpendicularly to the orientation of the nerve fibers (Supplementary Figure S1). Two animals, male identification (ID) 56 and female ID 77, were excluded from analysis due to the absence of visible thymic tissue in their sections, which would have compromised the consistency of diameter measurements.

### Quantification of total protein and cytokine levels

To ensure cytokine quantification was conducted within the required protein concentration (1 mg/mL), total protein concentrations in lung and brain homogenates were measured prior to the Luminex assay using the Pierce™ Bicinchoninic Acid (BCA) Protein Assay kit (23227, ThermoFisher Scientific), with bovine serum albumin as the standard. Absorbance was measured at 562 nm using a PowerWave XS Microplate spectrophotometer (BioTek).

A 9-Plex Multiplex Luminex Assay (Hamster Cytokine Panel-1, K22608, Ampersand Biosciences) was used to quantify a panel of cytokines and chemokines – IL-2, IL-4, IL-10, IL-6, IFN-γ, MCP-1, MIP-1α, TNF-α, and VEGF – in plasma, lung, and brain samples collected at 4, 32, and 60 dpi. Plasma samples were diluted 1:5 in sample dilution buffer, while brain and lung homogenates were diluted 1:10 and 1:15 in Mili-Q H_2_O, respectively, to achieve an estimated final protein concentration of 1 mg/mL. All samples were analyzed in triplicate according to the manufacturer’s instructions, and plates were read using the Luminex MagPix system (Luminex Corporation), equipped with RBM Plate Viewer software (v4.1.0.16979, Rules Based Medicine).

A hamster-specific ELISA kit (HMFI0011, Assay Genie) was used to quantify IL-1β in the same sample sets. Samples were diluted 1:2 in sample dilution buffer and analyzed in triplicate. Absorbance was measured at 450 nm on a PowerWave XS Microplate spectrophotometer (BioTek).

Results were normalized to total protein content and expressed as pg./mg protein for tissue homogenates and pg./mL for plasma samples.

### Neutralization antibody testing

Neutralizing activity in plasma was assessed in duplicate using the cPass SARS-CoV-2 Neutralization Antibody Detection Kit (L00847; GenScript), following the manufacturer’s instructions. Optical density (OD) at 450 nm was measured using a PowerWave XS Microplate spectrophotometer (BioTek). The assay included positive and negative controls provided by the manufacturer, which met all quality control criteria, as well as a SARS-CoV-2 neutralizing antibody calibrator (L00487; GenScript) for standard curve generation. Neutralizing activity was calculated as % inhibition = (1 − OD sample / OD negative control) × 100%, with values <30% considered negative, and antibody levels reported as semi-quantitative units/mL based on the standard curve. This assay was performed in all animals from both the negative control and SARS-CoV-2 groups at 4, 32, and 60 dpi, with the exception of one male in the SARS-CoV-2 group at 4 dpi due to insufficient plasma volume.

### Enzyme-linked immunospot (ELISpot) assay

The ELIspot assay was performed using splenocytes collected at 60 dpi with the hamster IFN-γ kit (3102-4APW-2, Mabtech) according to the manufacturer’s instructions. Briefly, cryopreserved splenocytes were quickly thawed and resuspended in RPMI 1640 (11875093, ThermoFisher Scientific) supplemented with 10% FBS (35-079-CV, Corning^®^), 1% glutamine, and 1% penicillin/streptomycin (all from Gibco Life Technologies). Cells were counted and plated at a concentration of approximately 2 × 10^5^ cells/100 μL/well in 96-well ELISpot plates. Cells were stimulated with either SARS-CoV-2 Spike protein (10 μg/mL; 40589-V08B1, Sino Biological), Nucleocapsid protein (10 μg/mL; 40588-V08B, Sino Biological), Concanavalin A (ConA, 7 μg/mL; L7647, Sigma-Aldrich™) as a positive control, and supplemented RPMI 1640 medium alone as a negative control, all in triplicate. Following 24 h of incubation at 37 °C, cells were removed, and membranes were developed according to the kit protocol to detect IFNγ spots. Spots were quantified using an AID iSpot Reader and AID ELISpot Software version 8.0 (Autoimmun Diagnostika GmbH). Assay validity was confirmed by a minimum of 100 spots per well in the ConA-stimulated positive control, with responses at least threefold higher than in unstimulated wells. Due to poor splenocyte isolation, the assay was successfully completed in 5 of 8 inoculated animals and 3 of 4 mock-inoculated animals euthanized at 60 dpi. ELISpot results were background-corrected by subtracting spot counts from paired unstimulated wells.

### Behavioral tests

Four neurocognitive tests—NBT, BT, OFT, and EZMT—were conducted at baseline (pre-inoculation) and during the post-acute phase of infection. These tests were chosen for their objective, quantifiable readouts, and demonstrated consistency across repeated trials (Tucker and McCabe, 2017). Tests were distributed over two consecutive days, progressing from least to most stressful. On day 1, animals underwent the BT in the morning, followed by the provision of a pressed cotton square (nestlet) for the NBT. On day 2, nests were scored in the morning, followed by the OFT, and, subsequently, the EZMT in the afternoon.

Due to the time-intensive nature of the behavioral tests and scheduling limitations within the BSL-3 facility, mock-inoculated controls (*n* = 4, two males and two females) were assessed on 17–18, 31–32, and 44–45 dpi, while SARS-CoV-2–inoculated hamsters (*n* = 8, four males and four females) were tested on 24–25, 38–39, and 58–59 dpi. Two-week intervals between sessions were implemented to minimize habituation and learning effects from repeated exposure (Deacon, 2012).

Hamsters were individually housed on minimal bedding (~2 cm high) in the procedure room 72 h prior to testing to facilitate acclimatization. All assays were performed under 70% dimmed lighting by the same observers. Following completion of testing on day 2, animals were returned to group housing. To minimize odors, the equipment was wiped with 5% ethanol between subjects and disinfected with PeraSafe™ between sessions.

For the BT, a PVC tube (68 mm in diameter, 200 mm in length, sealed at one end, with the open end raised 3 cm) containing 120 g of clay balls (8424084237824, Massó Garden) was placed inside each cage (Supplementary Video S1). After 30 min, the remaining material inside the tube was weighed following established protocols. This test assesses the natural burrowing behavior of rodents, which can be impaired by neurodegenerative deficit (Deacon, 2012).

In the NBT, a standard nestlet (NES3600, Ancare) was provided for 24 h without additional enrichment material (Supplementary Figure S2). Nests were scored on a five-point scale: 1 = untouched; 2 = < 50% shredded; 3 = 50–90% shredded without a distinct nest; 4 = > 90% shredded with material gathered into a flat nest; 5 = > 90% shredded forming a crater-shaped nest, as previously described. This test evaluates the rodents’ capacity to build nests, a behavior sensitive to neurodegenerative lesions (Deacon, 2012).

The OFT consisted in placing each hamster individually in the center of a white, opaque, square methacrylate arena (100 cm × 100 cm × 40 cm) and recording spontaneous behavior for 10 min with a high-resolution video camera (1080p 36 MP, IXNAIQY EU) (Supplementary Video S2). Videos were analyzed using Ethovision XT Basic Software (v17.0, Noldus Information Technology). This test assesses rodents’ natural aversion to large open spaces alongside their instinct to explore novel environments, providing insight into their emotional state and locomotor activity. Anxiety-related measures included the percentage of time spent in the center of the arena and the center-to-total distance ratio. Locomotor activity was evaluated by total distance moved (cm) and average velocity (cm/s) (Seibenhener and Wooten, 2015).

The EZMT involved placing each hamster individually in one of the open quadrants of a 100 cm-diameter annular platform elevated 50 cm above the floor, comprising two opposing enclosed (anxiolytic) and two opposing open (anxiogenic) quadrants (Panlab 760680). Animals were recorded for 10 min using a high-resolution video camera (1080p 36 MP, IXNAIQY EU) (Supplementary Video S3), and the videos were analyzed as described above. This test assesses rodents’ innate fear of heights and open spaces alongside their motivation to explore novel environments, providing insight primarily into their emotionality. Anxiety-related parameters were assessed based on the percentage of time spent and the distance moved (cm) within the open areas (Shepherd et al., 1994).

Delta (*Δ*) scores for each behavioral parameter were calculated by subtracting the pre-challenge (baseline) value from the post-challenge measurement; therefore, negative Δ values indicated a decline from baseline. This within-subject normalization accounted for inter-individual variability and allowed individual evaluation following SARS-CoV-2 inoculation. Additionally, z-scores were calculated to standardize Δ values by the negative control group mean and standard deviation, as previously described (Guilloux et al., 2011). For the BT, z-scores were normalized by sex but pooled across time points due to statistically significant sex differences without temporal effects, resulting in 6 control observations per sex. In contrast, the NBT, OFT, and EZMT Δ values were standardized using pooled control data across sexes and time points, as neither factor significantly affected performance, yielding 12 control observations per test.

### Statistical analyses

Data distributions were first assessed using the Shapiro–Wilk test for normality and F-test for homogeneity of variances. Body weight changes between groups were analyzed by a mixed-effects model with Šídák’s correction to account for repeated measures and group effects. Viral shedding data, which did not meet normality assumptions, were compared between sexes at each time point using the Kruskal-Wallis test with Dunn’s multiple-comparisons correction. The mean cross-sectional area (CSA) of the CVN was compared between groups using a two-tailed unpaired t-test, as the data were normally distributed with equal variances. Cytokine and behavioral data were analyzed by two-tailed unpaired t-tests when normally distributed with equal variances; Welch’s correction was applied if variances were unequal, and Mann–Whitney tests were conducted if normality was not met. For behavioral tests, z-scores for each parameter were calculated as previously described (Guilloux et al., 2011), with statistical significance determined at a threshold of ±1.96, assuming a normal distribution. All statistical analyses were conducted using GraphPad Prism v10.1.2, with significance set at α < 0.05.

## Results

### SARS-CoV-2 inoculation resulted in transient weight loss

**Figure 2:**
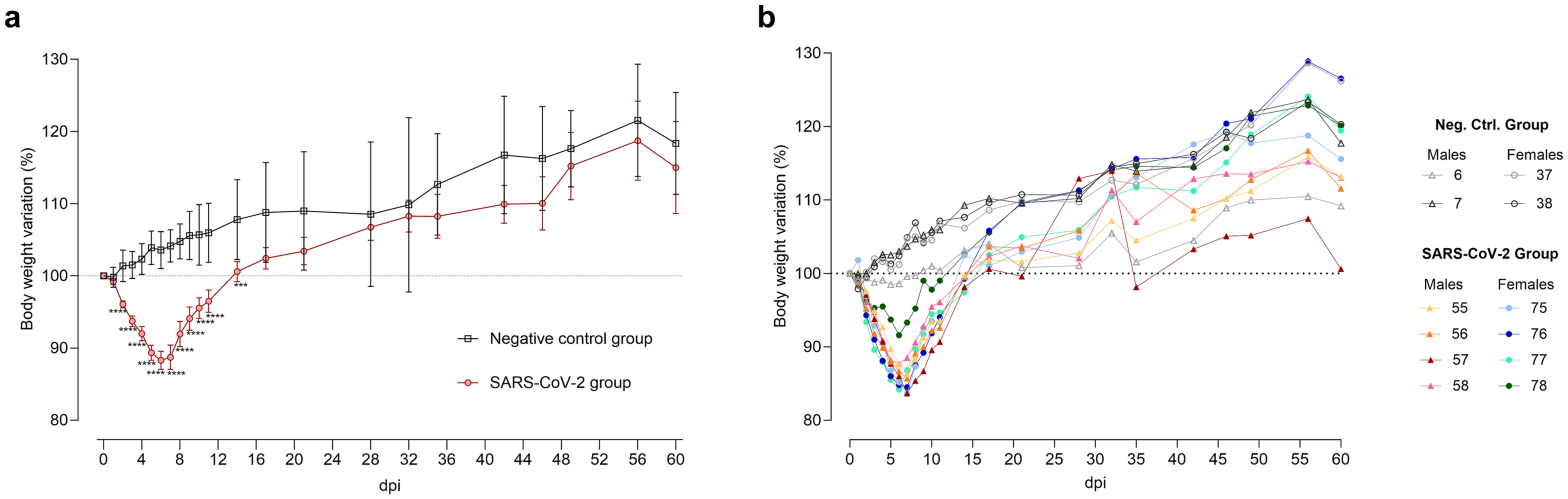
Body weight variation after SARS-CoV-2 inoculation. **(a)** Mean ± standard deviation of body weight variation in SARS-CoV-2-inoculated (circles, red) and negative control (squares, black) groups. Statistical significance, assessed by a mixed-effects model with Šídák’s correction, is indicated for *p* < 0.001 (***) and *p* < 0.0001 (****). **(b)** Individual body weight variation in hamsters euthanized at 60 days post-inoculation (dpi) for SARS-CoV-2-inoculated males (triangles, warm colors) and females (circles, cool colors), as well as negative control males (triangles, gray tones) and females (circles, gray tones).

SARS-CoV-2 inoculated hamsters (*n* = 40) exhibited gradual weight loss, reaching a mean reduction of 11.6% ± 3.8% standard deviation (SD) at 6 dpi. Weight recovery began at 7 dpi, with no significant differences between inoculated and control groups by 17 dpi (Figure 2a). When stratified by sex, weight loss peaked on average at 13.2% in males (*n* = 20) at 7 dpi, while females (*n* = 20) reached an average loss of 11.1% by 6 dpi, initiating recovery one day earlier. However, these sex-based differences were not statistically significant.

Heightened inter-individual weight loss variability was observed in the SARS-CoV-2 group during the acute phase (4–10 dpi) compared to controls, as indicated by higher coefficients of variation (CV). At 6 dpi, CV was 4.1% in inoculated males *versus* 2.2% in control males; at 7 dpi, 5.0% in inoculated females *versus* 2.9% in control females. By 60 dpi, variability converged across all groups. Notably, one male (ID 57) exhibited delayed recovery, consistent with a greater weight loss during the acute phase than other infected animals (Figure 2b).

### Sustained detection of SARS-CoV-2 gRNA in respiratory tissues throughout the study

**Figure 3:**
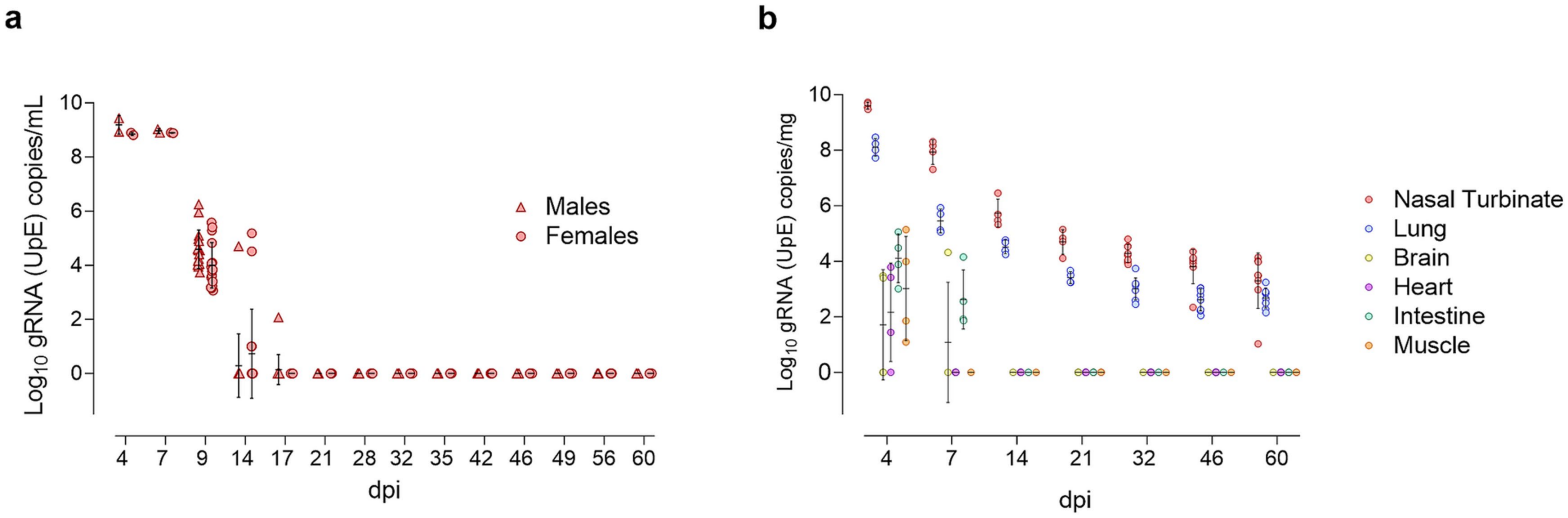
Longitudinal detection of SARS-CoV-2 gRNA in oropharyngeal swabs and tissues. **(a)** Log_10_ copy numbers of genomic RNA (gRNA) targeting the envelope protein gene (UpE) per milliliter of oropharyngeal swab supernatant, presented as individual data points for SARS-CoV-2-inoculated males (triangles, red) and females (circles, red), with means ± standard deviations. **(b)** Log_10_ gRNA (UpE) copy numbers per milligram of tissue, presented as individual data points for nasal turbinate (red), lung (blue), brain (yellow), heart (purple), intestine (green), and muscle (orange), with means ± SD.

In OS, SARS-CoV-2 gRNA loads peaked at 4 and 7 dpi, declined by 9 dpi, and became undetectable from 21 dpi (Figure 3a). All OS samples remained below the limit of quantification (<10^1.8^ TCID_50_/mL) in viral titration assays using Vero E6 cells at all time points.

In tissues, SARS-CoV-2 gRNA was detected across all samples at 4 dpi, with the highest concentrations in the nasal turbinate and lung, exceeding the levels in extra-respiratory tissues (intestine, muscle, heart, and brain) by more than 4 log units. By 7 dpi, gRNA remained detectable in both upper and lower respiratory tracts, as well as in the intestine, and brain. Viral gRNA persisted in respiratory tissues through 60 dpi, showing a gradual decline over time (Figure 3b). No significant differences in viral load were observed between males and females.

The highest infectious viral titers, measured by Vero E6 titration assays, were observed at 4 dpi in the nasal turbinate (log_10_ 5.93 TCID_50_/mL) and lung (log_10_ 4.51 TCID_50_/mL). At 7 dpi, only one nasal turbinate sample was positive, and no infectious virus was isolated from lung homogenates (Supplementary Figure S3). Infectious virus was not detected in extra-respiratory tissues at any time point (below the limit of quantification in all samples).

### Transient rhinitis and bronchointerstitial pneumonia induced by SARS-CoV-2 infection

In the nasal turbinate, mild to moderate (scores 1–2) inflammatory lesions were observed at 4 and 7 dpi, characterized by multifocal to diffuse muco-suppurative rhinitis (Supplementary Figure S4A). SARS-CoV-2 N protein levels were moderate to high (scores 2–3) at 4 dpi, and absent to minimal (scores 0–1) by 7 dpi (Supplementary Figure S4B). From 14 dpi onward, neither lesions nor N protein were detected.

In the lung, inflammatory lesions ranged from mild (n = 1) to moderate (n = 2) and severe (n = 1) at 4 dpi, characterized by multifocal peribronchial and peribronchiolar lymphohistiocytic infiltration, with type 2 pneumocyte hyperplasia and hypertrophy. Broncho-interstitial pneumonia peaked (score = 3) at 7 dpi, remitted to mild (score = 2) by 14 dpi, and resolved by 21 dpi. One animal displayed very mild pneumonia at 46 dpi, but this was not observed in samples examined at later time points (Supplementary Figure S4C). SARS-CoV-2 N protein levels in the lung were variable at 4 dpi, low at 7 dpi, and undetectable from 14 dpi onward (Supplementary Figure S4D).

No histopathological lesions or SARS-CoV-2 N protein were detected in extra-respiratory tissues (brain, heart, intestine, muscle) at any time post-inoculation.

### Increased variability in cervical vagus nerve cross-sectional area following SARS-CoV-2 inoculation

**Figure 4:**
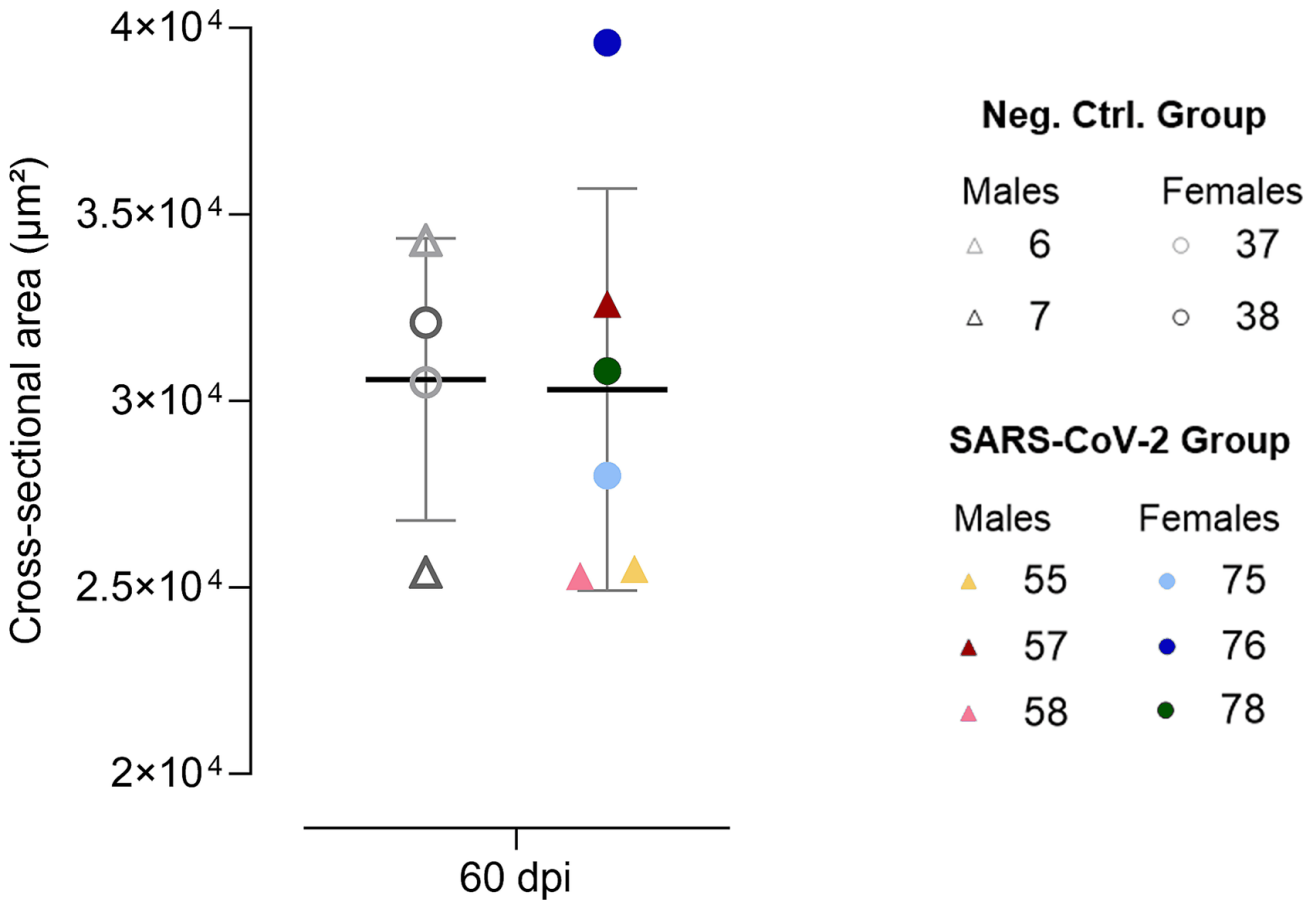
Cervical vagus nerve cross-sectional area at 60 days post-inoculation (dpi). Individual data points are shown for SARS-CoV-2-inoculated males (triangles, warm colors) and females (circles, cool colors), alongside negative control males (triangles, gray) and females (circles, gray), with means ± standard deviations represented.

At 60 dpi, one SARS-CoV-2 inoculated female (ID 76) exhibited a markedly increased CVN CSA. Although the SARS-CoV-2 group showed greater variability (25,300–39,600 μm^2^; CV = 18%) compared to controls (25,400–34,300 μm^2^; CV of 12%) (Figure 4), group comparison did not reveal statistically significant differences.

### Dynamic cytokine alterations in lung, brain, and plasma of SARS-CoV-2-inoculated hamsters

Cytokine profiling revealed pronounced time- and sex-dependent changes in the lung, brain, and plasma of SARS-CoV-2-inoculated hamsters. Across all compartments, infected animals displayed a sharp early increase in both pro- and anti-inflammatory mediators that gradually normalized but maintained notable individual and sex-specific variability.

**Figure 5:**
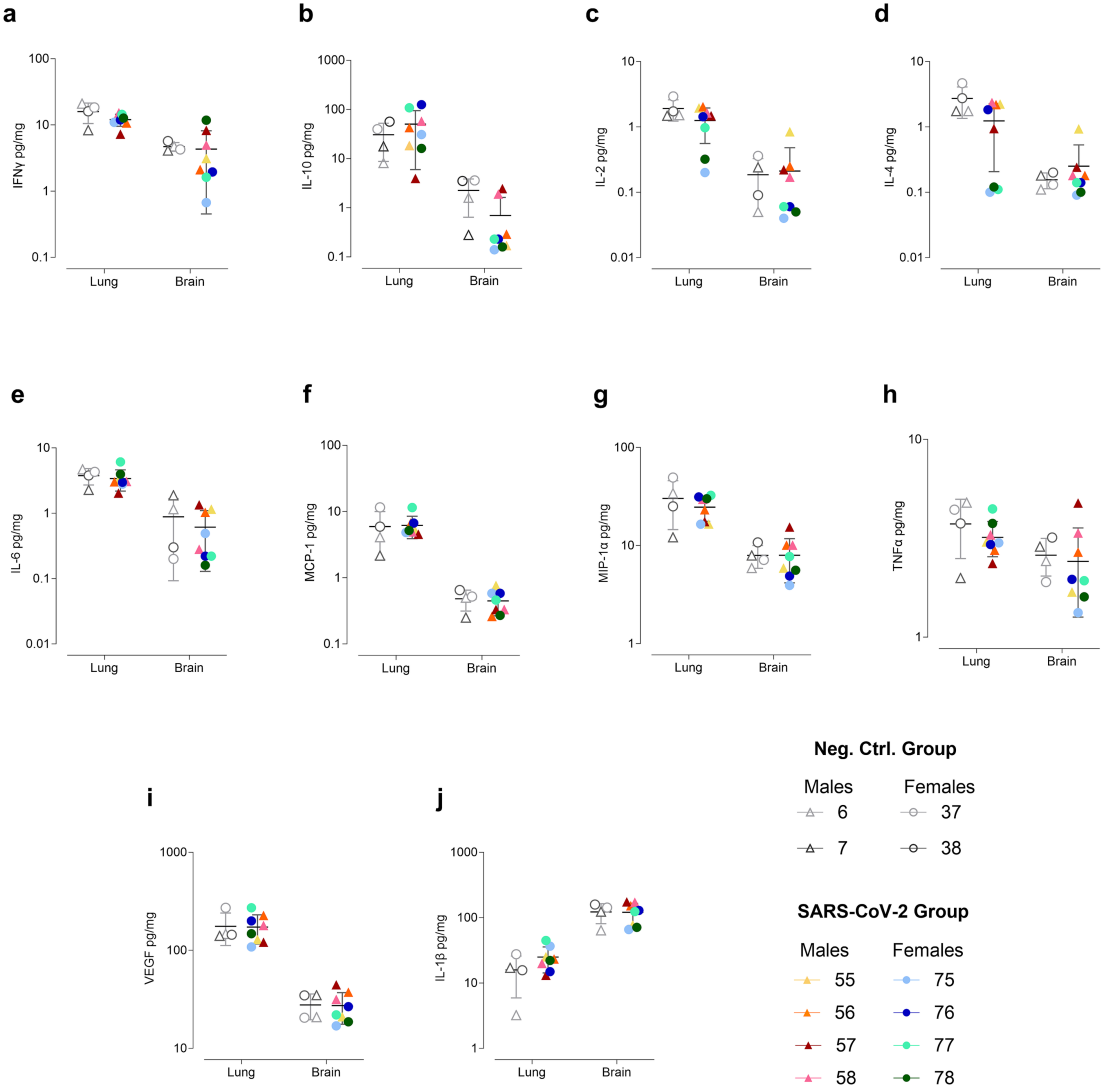
Cytokine concentrations in lung and brain at 60 days post inoculation (dpi). Cytokines measured include **(a)** IFNγ, **(b)** IL-10, **(c)** IL-2, **(d)** IL-4, **(e)** IL-6, **(f)** MCP-1, **(g)** MIP-1α, **(h)** TNFα, **(i)** VEGF, and **(j)** IL-1β. Concentrations (pg/mg protein) are shown on a logarithmic scale as individual data points for SARS-CoV-2-inoculated males (triangles, warm colors) and females (circles, cool colors), as well as negative control males (triangles, gray) and females (circles, gray), with the means ± standard deviations represented.

In the lung, both pro- and anti-inflammatory mediators increased significantly in infected animals by 4 dpi, including IL-10, IL-2, IL-4, MCP-1, MIP-1α, TNFα, IL-1β, and IFNγ. Notably, females exhibited IFNγ concentrations up to sixfold higher than males (Supplementary Figure S5). By 32 dpi, lung levels of IFNγ, IL-2, IL-4, MIP-1α, and IL-1β had fallen below control values, although two infected males and one female exhibited IL-10 levels threefold higher than controls. At 60 dpi, lung cytokine levels largely returned to baseline, except for elevated IL-10 and IL-1β and decreased IL-2 and IL-4 (Supplementary Figure S5). Specifically, IL-10 was twofold higher in two females (IDs 76, 77) and twofold lower in one male (ID 57) compared to controls. IL-1β was also elevated in two females (IDs 75, 77). IL-4 was reduced in one male (ID 57) and three females (IDs 75, 77, 78), with the latter additionally exhibiting decreased IL-2 (Figure 5).

In the brain, SARS-CoV-2-inoculated hamsters exhibited elevated levels of both pro- and anti-inflammatory cytokines at 4 dpi, including IFNγ, IL-10, IL-2, IL-4, IL-6, MCP-1, MIP-1α, TNFα, and IL-1β, compared to controls (Supplementary Figure S6). Notably, IFNγ, IL-10, IL-2, and IL-4 exhibited significantly high variability within the infected group, with females showing higher concentrations than males. By 32 dpi, IFNγ, IL-2, IL-4, MCP-1, MIP-1α, TNFα had declined in the SARS-CoV-2 group, while IL-10, VEGF, and IL-1β levels were comparable to controls. Additionally, sex-specific patterns were observed, with three females exhibiting the lowest IFNγ concentrations and consistently higher IL-1β concentrations compared to males (Supplementary Figure S6). At 60 dpi, mean cytokine levels in the SARS-CoV-2 group were generally comparable to controls, although IFNγ, TNFα, IL-10, and MIP-1α remained highly variable. Specifically, IFNγ levels were twofold higher in one male (ID 57) and one female (ID 78), while a twofold reduction was observed in another male (ID 56) and three females (IDs 75, 76, 77) (Figure 5).

Overall, cytokine concentrations in the brain were approximately one log lower than in the lung for all cytokines except for IL-1β, which was higher in the brain. Notably, at 4 dpi, MCP-1 was nearly 10,000-fold higher in the lung than in the brain, although this disparity diminished over time (Supplementary Figures S5, S6).

In plasma, cytokine levels were elevated in SARS-CoV-2-inoculated hamsters compared to controls at 4 dpi, including IFNγ, IL-10, IL-2, IL-4, IL-6, MCP-1, MIP-1α, TNFα, and IL-1β (Supplementary Figure S7). IFNγ and IL-1β levels were significantly elevated across all inoculated animals, while IL-10, IL-4, IL-6, and TNFα showed significant variability, with one male exhibiting the highest concentrations of all four cytokines. By 32 dpi, IL-2, IL-4, and IL-6 concentrations declined in the SARS-CoV-2 group, though individual variability remained high (Supplementary Figure S7). In contrast, MCP-1 and VEGF were elevated on average, with one male and three females showing marked increases, contributing to the heightened variability in the SARS-CoV-2 group (Supplementary Figure S7). At 60 dpi, plasma cytokine levels in inoculated hamsters were generally comparable to controls. However, four females (IDs 75–78) and one male (ID 58) exhibited IL-1β levels 10-fold higher than both controls and the other infected animals. Notably, one of these females (ID 78) also exhibited markedly elevated levels of IFNγ, IL-2, IL-4, IL-6, MIP-1α, and TNFα (Supplementary Figure S8).

### Sustained adaptive immune response post-SARS-CoV-2 inoculation

At 4 dpi, all animals showed low SARS-CoV-2 neutralizing activity, with females (*n* = 2) exhibiting higher levels (>100 U/mL) than males (<100 U/mL). By 32 dpi, robust neutralizing activity was observed in all animals, which was maintained up to 60 dpi. At these later time points, neutralizing antibody levels exceeded the assay’s upper limit of quantification (600 U/mL), indicating a durable and robust humoral immune response (Supplementary Figure S9A). No SARS-CoV-2 neutralizing antibodies were detected in the negative control at any time point (signal inhibition < 30%).

At 60 dpi, inoculated animals exhibited variable antigen-specific cellular immune responses, with IFNγ spot counts in response to Spike stimulation generally exceeding those elicited by Nucleocapsid. Female ID 76 showed the strongest response, with 109 spots/10^6^ cells for Spike and 51 spots/10^6^ cells for Nucleocapsid. Female ID 78 and male ID 57 mounted moderate Spike-biased responses, while male ID 58 and female ID 77 demonstrated the lowest IFNγ spot counts (Supplementary Figure S9B).

### A subset of animals underperformed in behavioral tests after SARS-CoV-2 inoculation

Analyses from all behavioral tests focused primarily on individual variability, given that group-level differences were not expected. To ensure consistency and comparability across subjects, results were normalized considering intra-individual variability, calculating individual changes from baseline (*Δ* scores). Δ scores were then standardized relative to the mean and standard deviation of the negative control group, expressed as z-scores. This approach allowed for a robust assessment of individual behavioral changes while minimizing the impact of baseline differences and inter-subject variability.

**Figure 6:**
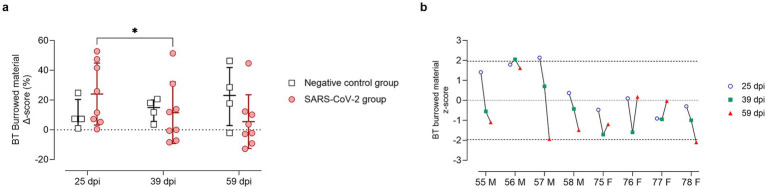
Burrowing test results at 25, 39, and 59 days post-SARS-CoV-2 inoculation. **(a)** Change in burrowed material (%) from baseline (Delta score, *Δ*) in SARS-CoV-2-inoculated animals (circles, red) and negative controls (squares, gray) at 25, 39, and 59 days post-inoculation (dpi). Individual data points are shown, with group means ± standard deviation. Statistical significance, determined using two-way ANOVA with Geisser–Greenhouse correction, is indicated for *p* < 0.05 (*). **(b)** Individual z-scores for burrowing performance in SARS-CoV-2-inoculated males (M, IDs 55–58) and females (F, IDs 75–78) at 25 (circles, blue), 39 (squares, green), and 59 (triangles, red) dpi. Z-scores indicate the number of standard deviations each observation deviates from the sex-matched control group mean. Dashed lines indicate the ± 1.96 z-score threshold for statistical significance; the dotted line represents a z-score of 0.

In the BT, SARS-CoV-2 inoculated GSH showed increased burrowing at 25 dpi compared to baseline and controls (Figure 6a). This increase was driven entirely by males, all with positive z-scores, whereas females scored at or below zero (Figure 6b). By 39 dpi, burrowing declined significantly (*p* < 0.05) compared to 25 dpi, returning to or dropping below baseline and control levels (Figure 6a), although one male (ID 56) maintained increased activity (Figure 6b). By 59 dpi, most females (IDs 75–77) trended toward recovery, except one (ID 78) that declined significantly. Conversely, most males (IDs 55, 57, 58) sustained reduced burrowing with negative z-scores (Figure 6b).

In the NBT, both SARS-CoV-2–inoculated and control animals exhibited high variability (Supplementary Figure S10A), with z-scores near zero at all time points (Supplementary Figure S10B).

**Figure 7:**
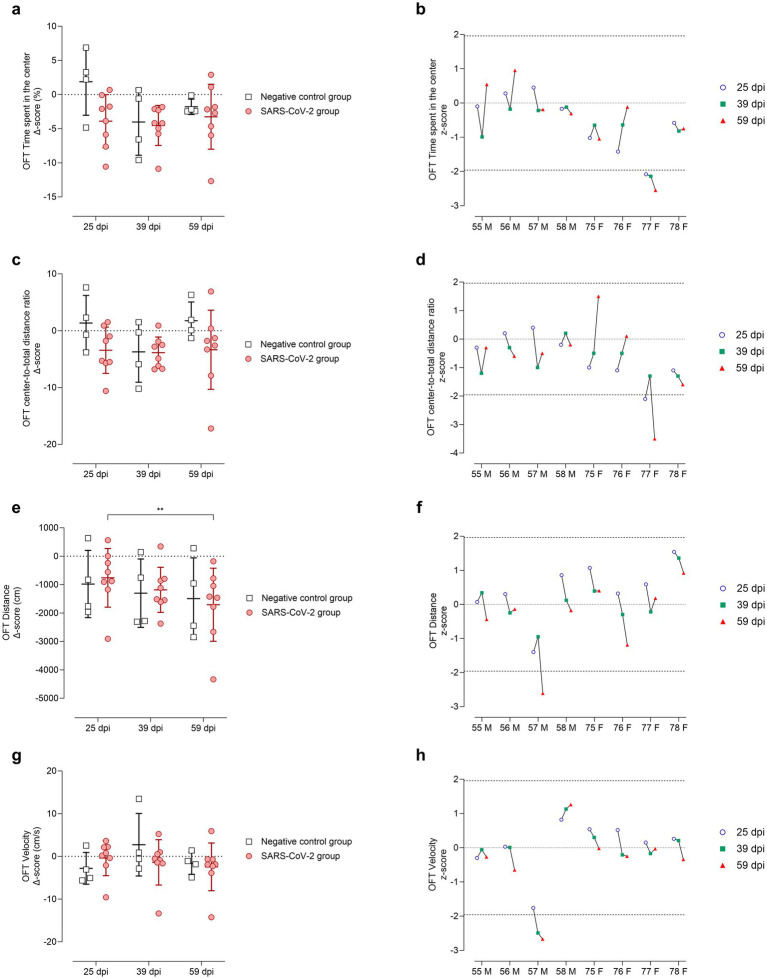
Open Field Test (OFT) performance at 25, 39, and 59 days post-SARS-CoV-2 inoculation. Panels **(a-b)** depict time spent in the inner zone, panels **(c-d)** center-to-total distance ratio, panels **(e-f)** total distance moved (cm), and panels **(g-h)** mean velocity (cm/s). **(a, c, e, g)** Changes from baseline (Δ scores) are shown for SARS-CoV-2-inoculated animals (circles, red) and negative controls (squares, gray) at 25, 39, and 59 days post-inoculation (dpi). Individual data points are shown with group means ± standard deviation. **(b, d, f, h)** Corresponding individual z-scores for SARS-CoV-2-inoculated males (M; IDs 55-58) and females (F; IDs 75-78) at 25 (circles, blue), 39 (squares, green), and 59 (triangles, red) dpi. Z-scores indicate the number of standard deviations each observation deviates from the control group mean. Dashed lines indicate the ±1.96 z-score threshold for statistical significance; the dotted line represents a z-score of 0.

In the OFT, anxiety-related behavior was assessed in terms of time spent in the center of the arena (Figures 7a,b) and center-to-total distance ratio (Figures 7c,d). At 25 dpi, SARS-CoV-2-inoculated animals spent less time in the center of the arena (Figure 7a) and reduced the center-to-total distance ratio (Figure 7c) compared to controls. This effect was driven entirely by females, whereas males showed no evident change from baseline (Figures 7b,d). By 39 dpi, group averages converged for both parameters; however, at 59 dpi, greater variability was observed within the SARS-CoV-2 group (Figures 7a,c). Notably, female ID 77 consistently spent less time in the center across all time points (*p* < 0.05, Figure 7b) and exhibited a reduced center-to-total distance ratio at 25 and 59 dpi (*p* < 0.05, Figure 7d).

Locomotor activity was evaluated based on total distance moved (Figures 7e,f) and velocity (Figures 7g,h). The SARS-CoV-2 group showed a progressive decline in distance traveled over time, with a statistically significant decrease between 25 and 59 dpi (*p* < 0.05, Figure 7e). Velocity remained comparable between SARS-CoV-2 and control groups (Figure 7g). Notably, male ID 57 exhibited a significant reduction in distance traveled at 59 dpi (*p* < 0.05, Figure 7f) and in velocity at both 39 and 59 dpi (*p* < 0.05, Figure 7h).

**Figure 8:**
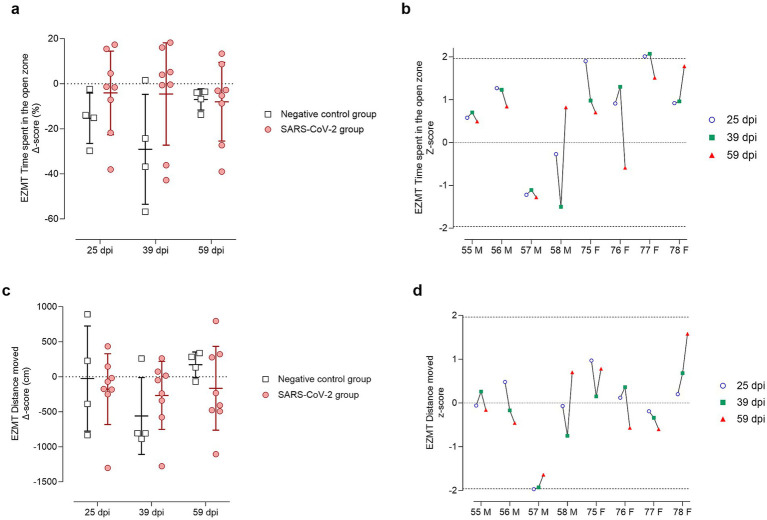
Elevated zero maze test (EZMT) results at 25, 39, and 59 days post-SARS-CoV-2 inoculation. Panels **(a,b)** show analyses of time spent in the open zone, while panels **(c,d)** show analyses of the distance (cm) moved within the open zone. **(a,c)** Change from baseline (Delta, Δ score) in SARS-CoV-2-inoculated animals (circles, red) and negative controls (squares, gray) at 25, 39, and 59 days post-inoculation (dpi). Individual data points are shown with group means ± standard deviation. **(b,d)** Corresponding individual z-scores for SARS-CoV-2-inoculated males (M; IDs 55–58) and females (F; IDs 75–78) at 25 (circles, blue), 39 (squares, green), and 59 (triangles, red) dpi. Z-scores indicate the number of standard deviations each observation deviates from the control group mean. Dashed lines indicate the ± 1.96 z-score threshold for statistical significance; the dotted line represents a z-score of 0.

In the EZMT, anxiety-like behavior was assessed by measuring time spent in the open arms (Figures 8a,b) and distance moved within this area (Figures 8c,d). At 25 dpi, infected animals generally increased open-zone exploration (Figure 8a), except for two males (IDs 57 and 58), which had negative z-scores (Figure 8b). Most infected animals maintained baseline activity (z-scores ≈ 0) for distance moved, except for male ID 57, who showed a significant reduction (Figure 8d). At 39 dpi, males IDs 57 and 58 again demonstrated reduced open-zone time and movement (Figures 8b,d). By 59 dpi, male ID 58 returned to baseline, while male ID 57 and female ID 76 underperformed on both measures (Figures 8b,d).

A comprehensive analysis of all behavioral tests using z-score standardized data is presented in Supplementary Figures S11, S12. At 25 dpi, male ID 57 showed reduced performance in both the OFT and EZMT (Supplementary Figure S11A), while all four females (IDs 75–78) underperformed in the OFT (Supplementary Figure S12A). By 39 dpi, male performance declined further, with three out of four individuals (IDs 55, 57, 58) exhibiting deficits across multiple tests (Supplementary Figure 11B). All females (IDs 75–78) continued to underperform in the OFT and began to reduce performance in the BT (Supplementary Figure S12B). At 59 dpi, male ID 57 again displayed the lowest scores across nearly all behavioral measures (Supplementary Figure S11C), and males IDs 55 and 58 continued to underperform in the BT. Female outcomes varied across the four tests, but their behavior in the OFT was consistently compromised, with scores lower than those of the control group (Supplementary Figure S12C).

Overall, males exhibited greater impairments in the BT and EZMT, while females demonstrated consistent deficits in the OFT, suggesting potential sex-specific patterns of behavioral dysfunction. Notably, male ID 57 consistently underperformed in both the EZMT and OFT across all time points, as well as in the BT from 39 dpi onward (Supplementary Figure S11). Similarly, female ID 77 displayed persistent deficits in the OFT throughout the study period (Supplementary Figure S12).

## Discussion

Since the WHO declared COVID-19 a pandemic in March 2020, translational animal models have been instrumental in elucidating the disease pathogenesis and evaluating the efficacy of prophylactic and therapeutic interventions. Among these, GSH models have been extensively characterized and widely used due to their ability to recapitulate a mild-to-moderate, human-like acute illness (Usai et al., 2023). However, their potential to model the neurological symptomatology of PCC in humans remains largely underexplored.

GSH have emerged as a potential preclinical model for PCC, encompassing a range of sensory, pulmonary, immunological, cardiovascular, muscular, and metabolic sequelae (Frere et al., 2022; Hsu et al., 2022; Mulka et al., 2022; Rizvi et al., 2022; Serafini et al., 2023; Boese et al., 2024; Homma et al., 2024). Here, we report the longest evaluation of SARS-CoV-2-induced neurological outcomes in GSH to date, extending up to 60 dpi. Considering that approximately 2 weeks in hamsters correspond to one human year (Dutta and Sengupta, 2019), the current 60-day observation period represents a substantially prolonged timeframe for evaluating potential long-term viral sequelae. To maximize the likelihood of inducing PCC-like phenotypes, we employed the ancestral SARS-CoV-2 strain with the D614G mutation, which elicits a more severe acute pathology in GSH compared to later variants (Davis et al., 2021; Abdelnabi et al., 2022). This is particularly relevant, as greater severity and higher viral loads during the acute phase of infection in humans have been associated with an increased risk of developing persistent post-COVID-19 symptoms. However, it is important to note that PCC can also develop following mild or even asymptomatic infections (Malkova et al., 2021; Peluso and Deeks, 2024; Herbert et al., 2025).

During acute infection, weight loss paralleled viral shedding, the presence of infectious virus, and histopathological lesions in respiratory tissues, as well as low levels of viral gRNA without associated lesions in non-respiratory sites, consistent with previous reports (Sia et al., 2020). Notably, gRNA detection in the brain coincided with elevated neuroinflammatory cytokines, particularly in females, mirroring neurological signs and cognitive-dysfunction predictors identified in human PCC (Ding and Zhao, 2023; Müller and Di Benedetto, 2024). Additionally, respiratory and systemic inflammation were observed, consistent with human studies that link elevated cytokine levels to greater disease severity (Liu et al., 2020). Importantly, sex-related differences were evident: males showed greater weight loss and comparatively weaker cytokine responses in the brain and lung, whereas females mounted stronger pulmonary IFNγ responses, in agreement with higher immune responses observed in women during acute viral infections (Takahashi et al., 2020).

In the post-acute phase, animals regained weight, and no infectious virus was further detected, as reported in prior GSH studies (Frere et al., 2022). Nonetheless, viral gRNA persisted in respiratory tissues during 60 dpi, in line with proposed mechanisms of PCC in humans (Antar et al., 2023; Peluso and Deeks, 2024). Neuro-PCC-like deficits were evaluated through the NBT and BT, which evaluated innate behaviors analogous to human “activities of daily living” (Deacon, 2012), and the OFT and EZMT, which measured general locomotor activity and anxiety-related emotional behaviors, paralleling executive dysfunction and depressive or anxious phenotypes common in PCC patients (Shepherd et al., 1994; Seibenhener and Wooten, 2015; Mina et al., 2023). These behavioral tests were selected for their objective, quantifiable outcomes and demonstrated consistency across repeated trials, with two-week intervals minimizing habituation and learning effects due to over-exposure (Deacon, 2012; Tucker and McCabe, 2017). While these assays are well established in rats and mice, their application in hamsters is relatively uncommon, underscoring the novelty of this approach. However, prior studies have demonstrated their successful implementation and reproducibility in GSH (Swanson, 1966; Deacon, 2009; Frere et al., 2022). Although the NBT did not detect SARS-CoV-2-related deficits, the OFT, EZMT, and BT consistently identified neurocognitive impairments in a subset of animals, suggesting deficits in “activities of daily living,” exploratory behavior and stress coping (Shepherd et al., 1994; Deacon, 2012; Seibenhener and Wooten, 2015). Among males with behavioral deficits, IDs 57 and 58 had the highest levels of TNF-α in the brain, a hallmark of neuroinflammation, tissue damage, and neurodegeneration associated with PCC (Domingues et al., 2017; Del Valle et al., 2020; Devlin and Gombolay, 2023; Peluso and Deeks, 2024). Both also showed relatively elevated IL-10 in brain, potentially reflecting a compensatory anti-inflammatory response to mitigate neural damage (Kamaltdinova et al., 2021). Male ID 58 displayed increased plasma IL-1β, a cytokine commonly upregulated in PCC patients (Woodruff et al., 2023). Notably, male ID 57, which demonstrated the most severe behavioral deficits, also exhibited elevated brain levels of MIP-1α and IFN-γ, cytokines linked to ischemic events, neurodegeneration, and cognitive dysfunction (Marciniak et al., 2015; Janach et al., 2020). Among females with behavioral impairments, IDs 76, 77, and 78 showed reduced brain IL-2, which may impair immune regulation and contribute to persistent inflammation or neurodegeneration (Poletti et al., 2024). All three also exhibited elevated plasma IL-1β, consistent with inflammatory signatures observed in PCC patients (Woodruff et al., 2023). Additionally, two females (IDs 77 and 78) displayed reduced lung IL-4 expression, potentially impairing tissue repair (Allen, 2023), while elevated IL-10 in females IDs 76 and 77 may indicate immune exhaustion following intense immune activation (Carlini et al., 2023; Peluso and Deeks, 2024). In particular, female ID 78 displayed elevated plasma levels of multiple cytokines (IFN-γ, IL-2, IL-4, IL-6, MIP-1α, TNF-α, and IL-1β), along with increased brain IFN-γ levels. This profile suggests a systemic inflammatory response that may promote neuroinflammation and contribute to cognitive deficits (Janach et al., 2020).

The variability in cytokine profiles and behavioral outcomes observed during the post-acute phase mirrors the complexity of human PCC, with animal subpopulations resembling proposed clinical subtypes. For instance, animals such as IDs 57 and 78, which exhibited reduced burrowing activity, may reflect a cognitive-dominant, neuroinflammatory subtype, while hamster ID 77, which exhibited anxiety-like behavior, parallels a mental health-dominant subtype with immune dysregulation (Ozonoff et al., 2024; Peluso and Deeks, 2024). Notably, GSH with evident behavioral disorders showed reduced exploration and heightened anxiety-like features, suggesting overlapping symptom domains. Although not directly assessed in this study, GSH are known to develop acute-phase anosmia with gradual recovery and persistent individual variability at 42 dpi (Reyna et al., 2022), potentially modeling a neurosensorial PCC phenotype.

Among animals exhibiting both behavioral and cytokine alterations, long-term virological, and cellular immune and histopathological changes were also observed. For instance, female ID 76 exhibited the highest lung viral RNA load at 60 dpi, a strong IFNγ cellular response, and increased CVN CSA. Individual-level analyses further suggest that acute-phase severity and viral burden may contribute to long-term behavioral deficits. For example, male ID 57 exhibited the greatest weight loss during the acute phase, and female ID 76 displayed the highest viral shedding in OS at 9 dpi. These findings align with human data linking greater acute severity and delayed viral clearance to higher PCC risk. However, as in humans, there is substantial individual variability, and this association is not uniformly predictive across the cohort (Peluso and Deeks, 2024; Herbert et al., 2025).

The development of PCC is thought to result from a multifactorial interplay of pathophysiological mechanisms, including viral persistence (either SARS-CoV-2 or its components), chronic inflammation and immune dysregulation, autoimmunity, coagulopathy, gut dysbiosis, mitochondrial dysfunction and oxidative stress, metabolic dysregulation, and/or dyasautonomia. Our findings support the value of the GSH model in recapitulating several of these key features of the human PCC, including: (1) persistent viral gRNA, (2) dysregulated immune responses, and (3) structural alterations in the vagus nerve, each of which potentially contributing to the observed neurocognitive impairments (Antar et al., 2023; Davis et al., 2023; Lladós et al., 2024; Peluso and Deeks, 2024).

In this study, the presence of persistent SARS-CoV-2 RNA without detectable infectious virus beyond 7 dpi suggests that the viral gRNA likely represents remnants of a resolved infection. However, low-level viral replication below the TCID_50_ detection threshold (<10^1.8^ TCID_50_/mL) cannot be completely ruled out. Importantly, even non-replicating viral remnants may act as persistent antigenic stimuli, capable of sustaining systemic immune dysregulation (Hoagland et al., 2021).

Consistent with this, immune dysregulation was observed at 60 dpi, characterized by elevated IL-1β and IFNγ levels in animals with behavioral impairments. These circulating cytokines may reach the brain via humoral routes, particularly if blood–brain barrier integrity is compromised, or through vagus nerve signaling (Dedoncker et al., 2021; Greene et al., 2024). Upon reaching the CNS, pro-inflammatory mediators can disrupt neurotransmitter systems and neural circuits, particularly those involving the basal ganglia and anterior cingulate cortex, which are essential for motor function, motivation, and emotional regulation (Miller et al., 2013). In fact, it is known that low-grade cytokine elevations can disrupt the metabolism of neurotransmitters such as serotonin, dopamine, and glutamate, contributing to cognitive and behavioral deficits (Pollmächer et al., 2002; Monteiro et al., 2016; Dedoncker et al., 2021).

Additionally, structural alterations in the vagus nerve were identified in a single animal (ID 76). While this isolated finding limits definitive conclusions, it is a notable finding considering the heterogeneity of human PCC and previous reports of vagus nerve involvement (Lladós et al., 2024; Peluso and Deeks, 2024). This vagal alteration may represent a meaningful signal warranting further study in larger cohorts, ideally supported by expanded autonomic and neuroimaging assessments.

Overall, the GSH model demonstrates several strengths in recapitulating key features of PCC. Given that PCC affects only a subset of individuals and manifests across diverse clinical phenotypes (Ozonoff et al., 2024; Peluso and Deeks, 2024), a similarly heterogeneous response was expected. By adopting an individual-level analytical approach rather than conventional group-based statistics, we identified subject-specific alterations in cytokine profiles and behavioral outcomes during the post-acute phase. This approach also revealed nuanced associations between acute-phase severity and long-term outcome. The coexistence of immunological, virological and behavioral alterations in the absence of direct neuroinvasion supports the utility of this model for studying neuroimmune mechanisms underlying PCC. However, some limitations should be also acknowledged. First, the model currently lacks validated cognitive tests for assessing memory or executive function, as tasks like novel object recognition are confounded by rapid habituation and learned avoidance behaviors in GSH (Reyna et al., 2025). Second, immune profiling was restricted by the limited availability of species-specific reagents and the inability to perform longitudinal analyses within the same animals due to logistical challenges in the BSL-3 facilities. Third, the sample size hindered the identification of PCC subtypes, sex-related differences, and specific biomarkers, as only a subset of animals exhibited measurable alterations, reflecting the clinical heterogeneity of PCC, where persistent symptoms develop only in a proportion of infected individuals.

This study provides a basis for future research into the underlying mechanisms and emphasizes the potential benefits of targeting cytokines for therapeutic intervention. Larger cohorts will be essential to more robustly define and validate PCC phenotypic clusters, explore sex-related differences, and establish causal links between cytokine and behavioral alterations. This animal model could also be leveraged to analyze other pathological mechanisms implicated in PCC, including autoimmunity, coagulopathy, gut dysbiosis, mitochondrial dysfunction, and metabolic dysregulation. Given the complexity and multifactorial nature of PCC, complementary animal models will be needed to capture its full spectrum. Although translational limitations exist, such as species-specific differences in lifespan, viral kinetics, genetic background, and aging processes, animal models remain essential for elucidating PCC mechanisms and advancing biomarker discovery and therapeutic development.

## Data Availability

The original contributions presented in the study are included in the article/Supplementary material, further inquiries can be directed to the corresponding authors.
