# Supplementary material for: Long-term neurocognitive outcomes of SARS-CoV-2 infection in a hamster model
Source: Front Microbiol. 2025 Aug 14;16:1646616. doi: 10.3389/fmicb.2025.1646616 (PMC12390999; doi:10.3389/fmicb.2025.1646616)
Supplement: Supplementary file 4 [file Data_Sheet_1.docx]

Supplementary Material

# Supplementary Figures and Videos

## Supplementary Figures


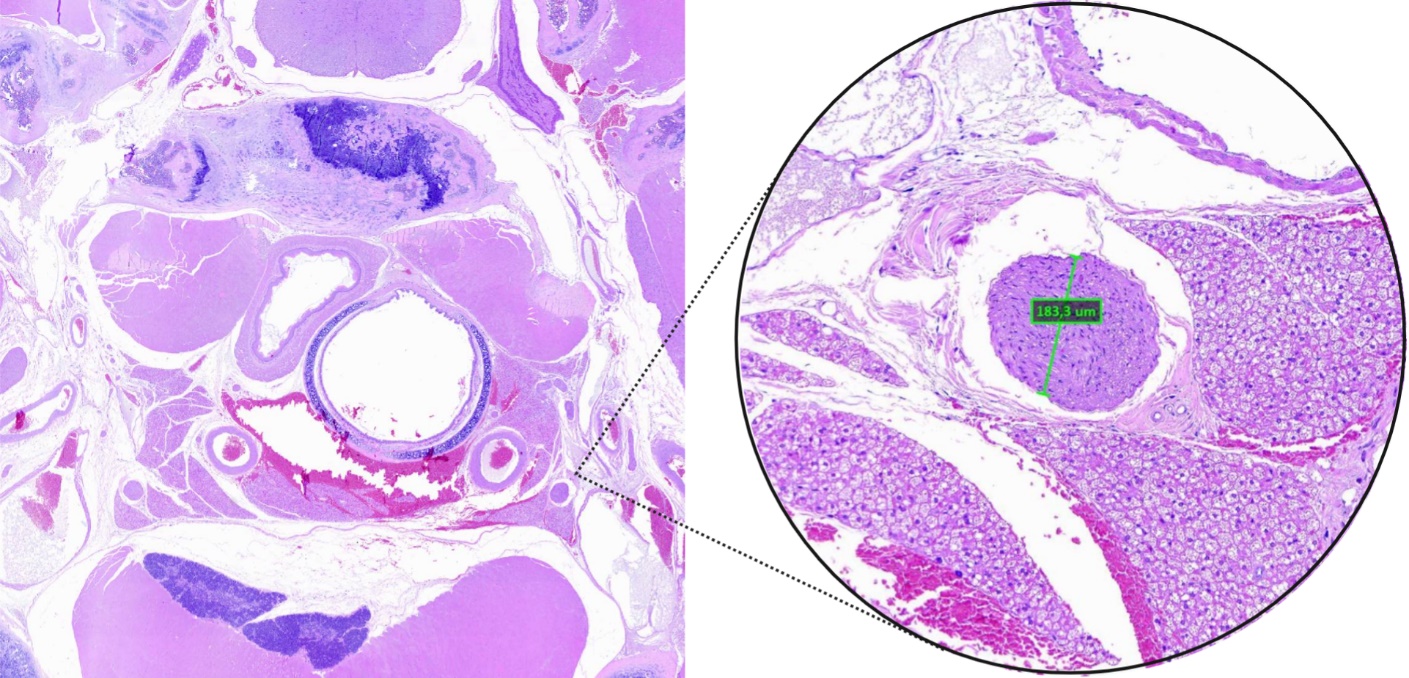


**Supplementary Figure S1.** Hematoxylin and eosin-stained cross-section of the cervical vagus nerve. The left panel provides an overview of cervical anatomical structures, including the thymus (asterisk), with the cervical vagus nerve indicated. The right panel displays a magnified view of the vagus nerve cross-section, featuring the diameter measurement (183.3 µm in this example), taken perpendicular to the orientation of the nerve fibers.


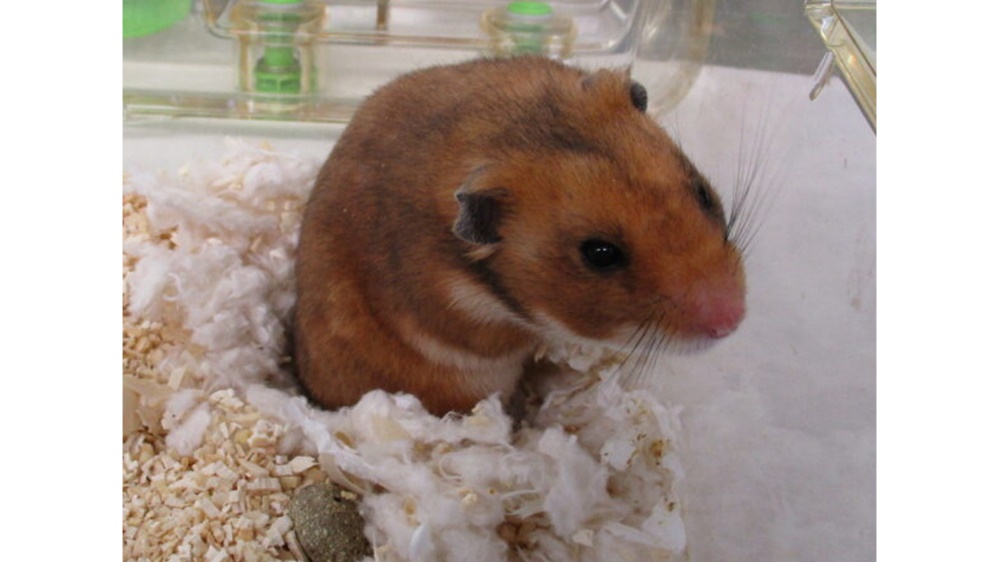


**Supplementary Figure S2.** Nest construction in the nest building test.

_
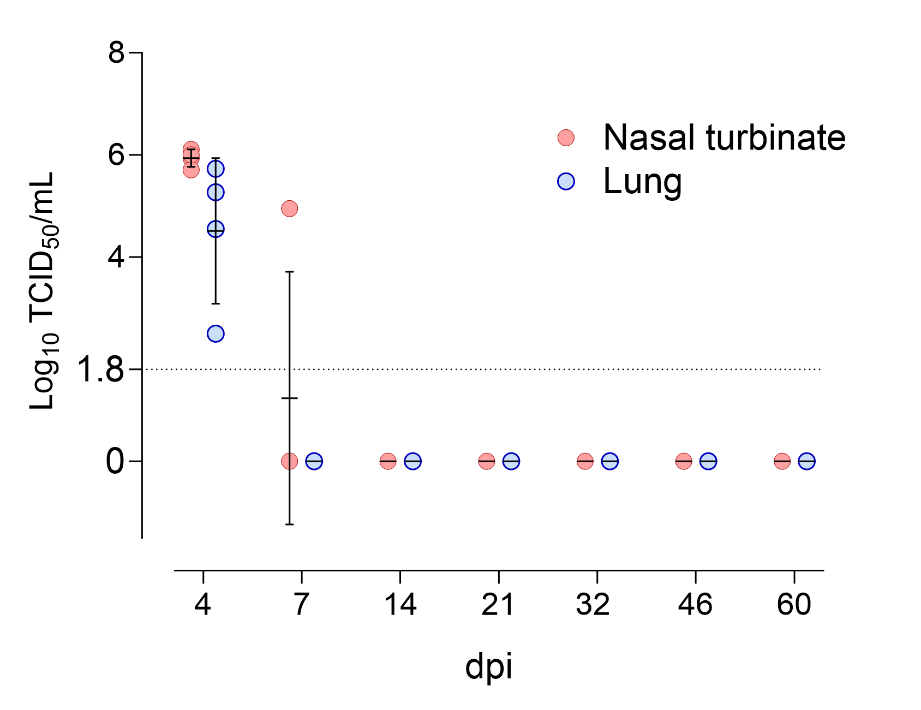
_

**Supplementary Figure S3.** Infectious viral titres in respiratory sites. Infectious viral titres reported as log10 Tissue Culture Infectious Dose 50% (TCID)/mL, presented as mean ± SD, with individual data points for nasal turbinate (red) and lung (blue). The dotted line represents the limit of quantification (101.8 TCID_50_/mL).





**Supplementary Figure S4. (A)** Histopathology and **(B)** immunohistochemistry (IHC) scores in the nasal turbinate, along with **(C)** histopathology and **(D)** IHC scores in the lung at 4, 7, 14, 21, 32, 46, and 60 days post-inoculation (dpi). Data are shown as individual points with means ± standard deviations. Histopathological lesions in **(A)** and **(C)** were scored semi-quantitatively on a 0-3 scale: 3: 0 = none, 1 = mild, 2 = moderate, and 3 = severe. IHC detection of nucleoprotein antigen in **(B)** and **(D)** was scored semi-quantitatively on 0-3 scale: 0 = none, 1 = low, 2 = moderate, and 3 = high.


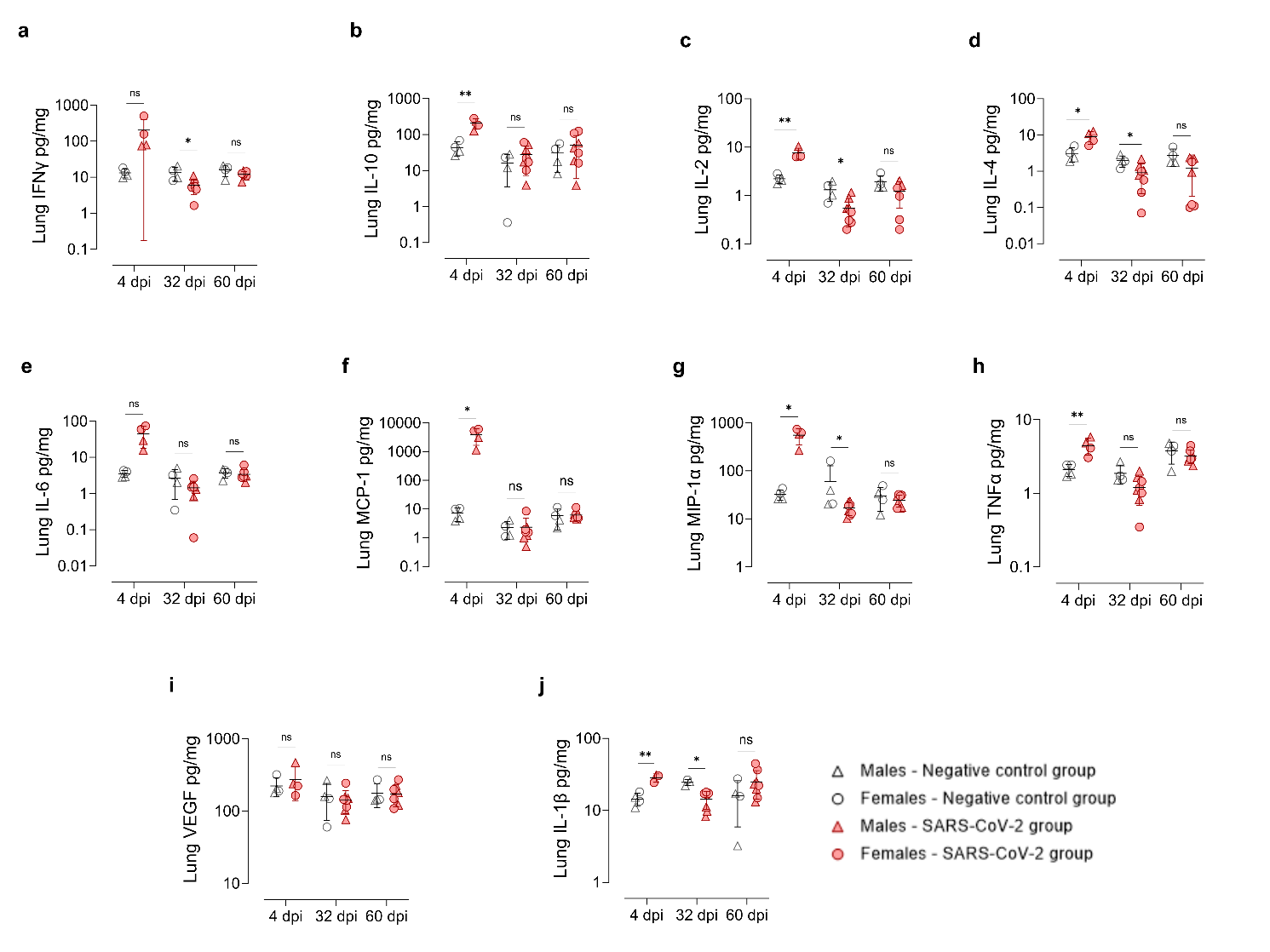


**Supplementary Figure S5.** Cytokine concentrations in the lung at 4, 32 and 60 days after SARS-CoV-2 inoculation. Cytokines measured in the lung include **(A)** IFNγ, **(B)** IL-10, **(C)** IL-2, **(D)** IL-4, **(E)** IL-6, **(F)** MCP-1, **(G)** MIP-1α, **(H)** TNFα, **(I)** VEGF, and **(J)** IL-1β. Concentrations (pg/mg protein) at 4, 32, and 60 days post-inoculation (dpi) are shown on a logarithmic scale as individual data points for SARS-CoV-2-inoculated males (triangles, red) and females (circles, red), as well as negative control males (triangles, black) and females (circles, black), with means ± standard deviations indicated. Statistical significance, determined by two-tailed unpaired *t*-test or by two-tailed Mann-Whitney tests, is shown for *p* < 0.05 (*) and *p* < 0.01 (**).


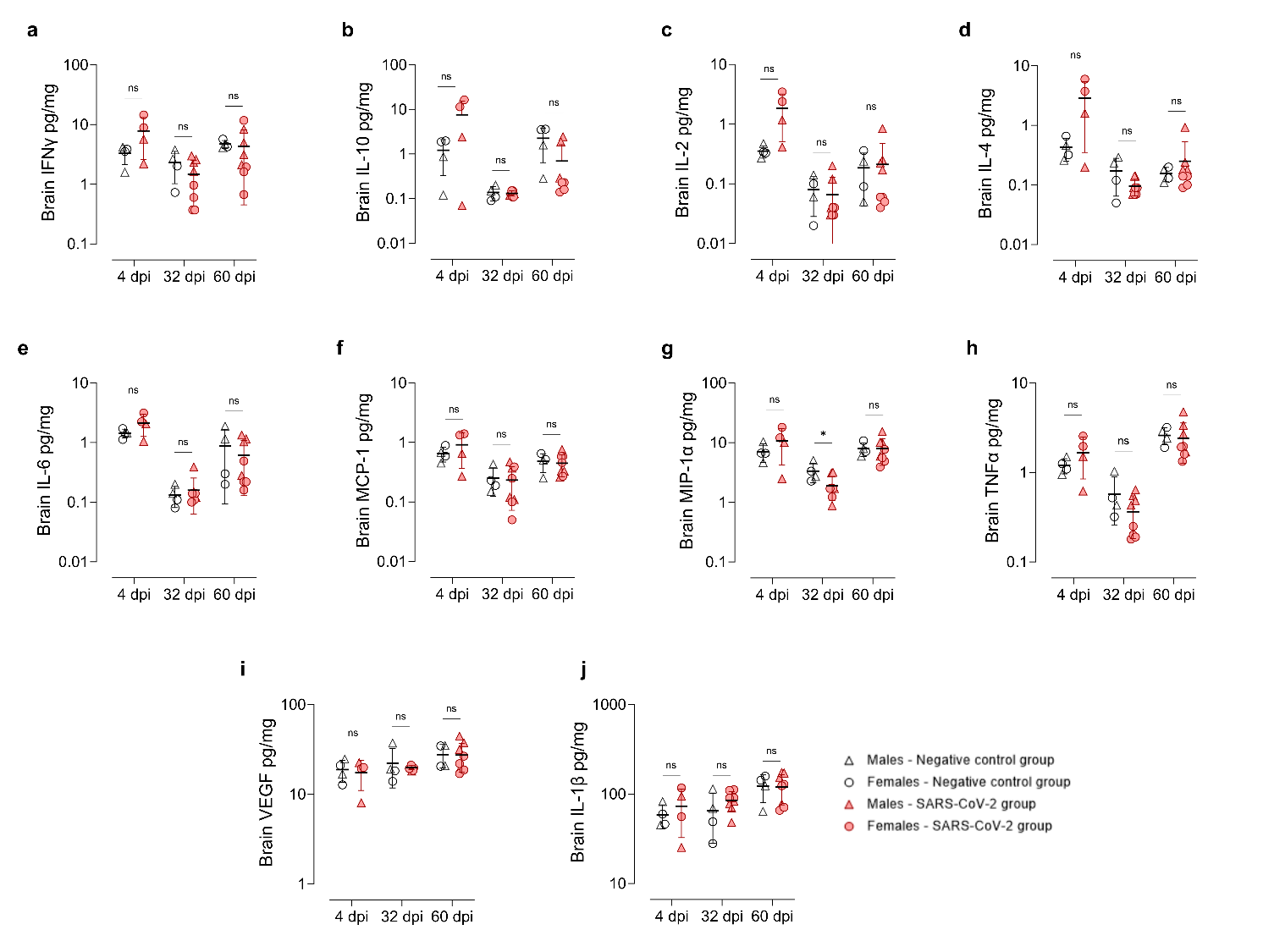
 **Supplementary Figure S6.** Cytokine concentrations in the brain at 4, 32 and 60 days after SARS-CoV-2 inoculation. Cytokines measured in the brain include **(A)** IFNγ, **(B)** IL-10, **(C)** IL-2, **(D)** IL-4, **(E)** IL-6, **(F)** MCP-1, **(G)** MIP-1α, **(H)** TNFα, **(I)** VEGF, and **(J)** IL-1β at 4, 32, and 60 days post-inoculation (dpi). Concentrations (pg/mg protein) are shown on a logarithmic scale as individual data points for SARS-CoV-2-inoculated males (triangles, red) and females (circles, red), as well as negative control males (triangles, black) and females (circles, black), with means ± standard deviations indicated. Statistical significance, determined by two-tailed unpaired t-test or by two-tailed Mann-Whitney tests, is shown for p < 0.05 (*).


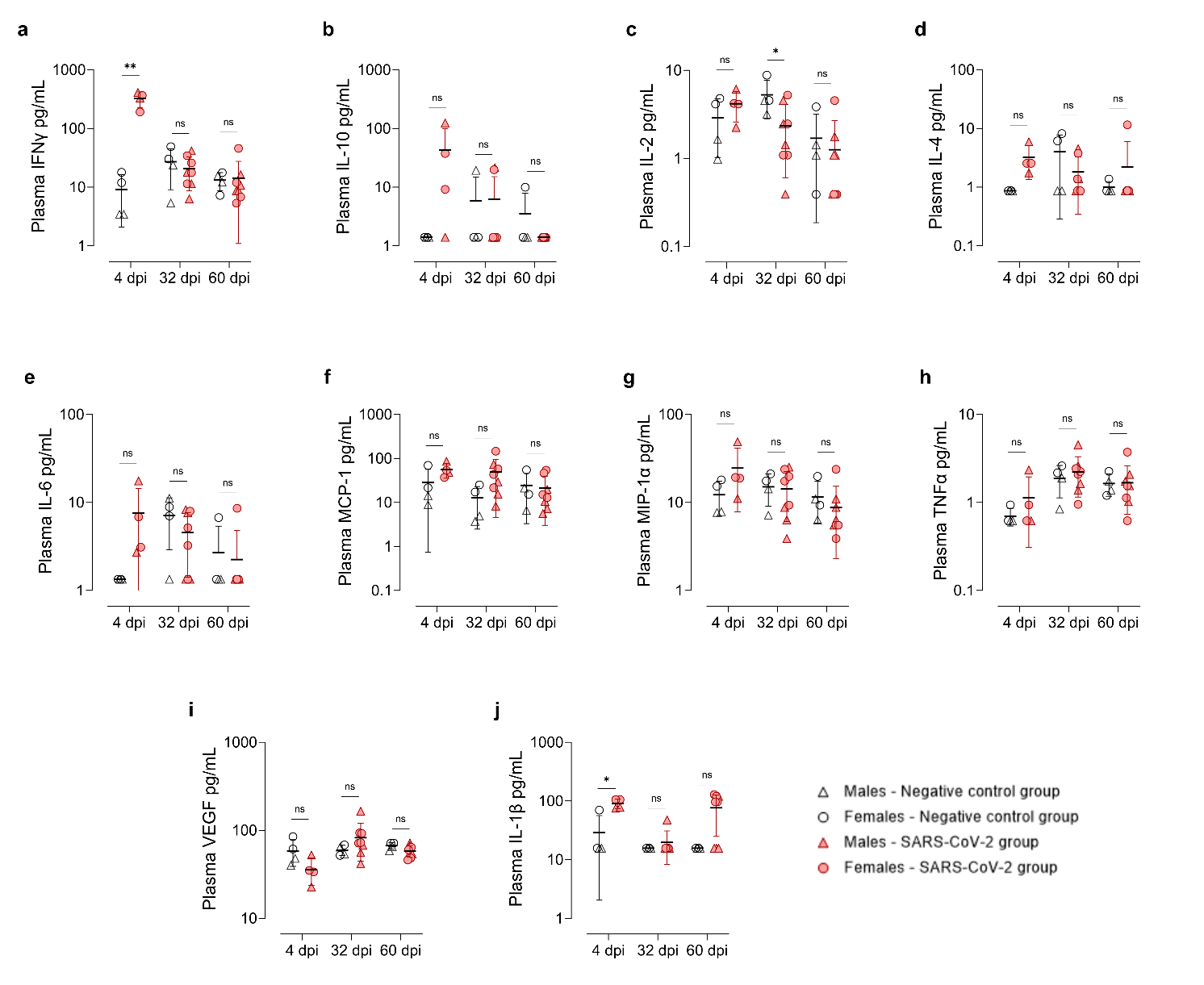


**Supplementary Figure S7.** Cytokine profile in plasma at 4, 32 and 60 days after SARS-CoV-2 inoculation in GSH**.** Cytokines measured include **(A)** IFNγ, **(B)** IL-10, **(C)** IL-2, **(D)** IL-4, **(E)** IL-6, **(F)** MCP-1, **(G)** MIP-1α, **(H)** TNFα, **(I)** VEGF, and **(J)** IL-1β at 4, 32, and 60 dpi. Cytokine concentrations are expressed as picograms per milliliter of plasma (pg/mL) and are presented as mean ± standard deviation with individual data points for SARS-CoV-2-inoculated males (triangles, red) and females (circles, red), as well as negative control males (triangles, black) and females (circles, black) on a logarithmic scale. Statistical significance is indicated for p-values < 0.05 (*) and < 0.01 (**) between the inoculated and control groups.


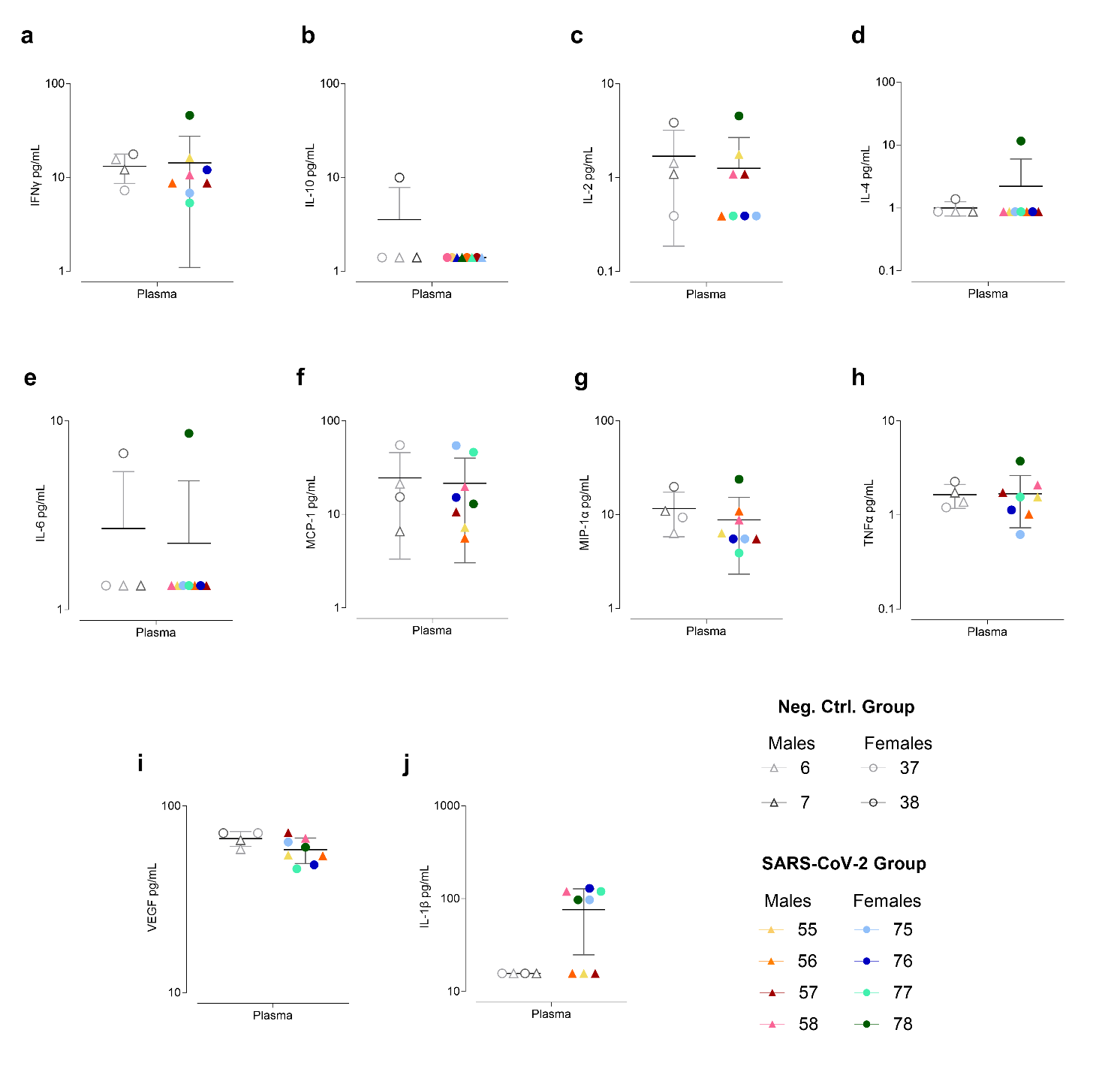


**Supplementary Figure S8.** Cytokine concentrations in plasma at 60 days post-inoculation. Cytokines measured include **(A)** IFNγ, **(B)** IL-10, **(C)** IL-2, **(D)** IL-4, **(E)** IL-6, **(F)** MCP-1, **(G)** MIP-1α, **(H)** TNFα, **(I)** VEGF, and **(J)** IL-1β. Concentrations (pg/mL plasma) are shown on a logarithmic scale as individual data points for SARS-CoV-2-inoculated males (triangles, warm colors) and females (circles, cool colors), alongside negative control males (triangles, grey) and females (circles, grey), with means ± standard deviations indicated.


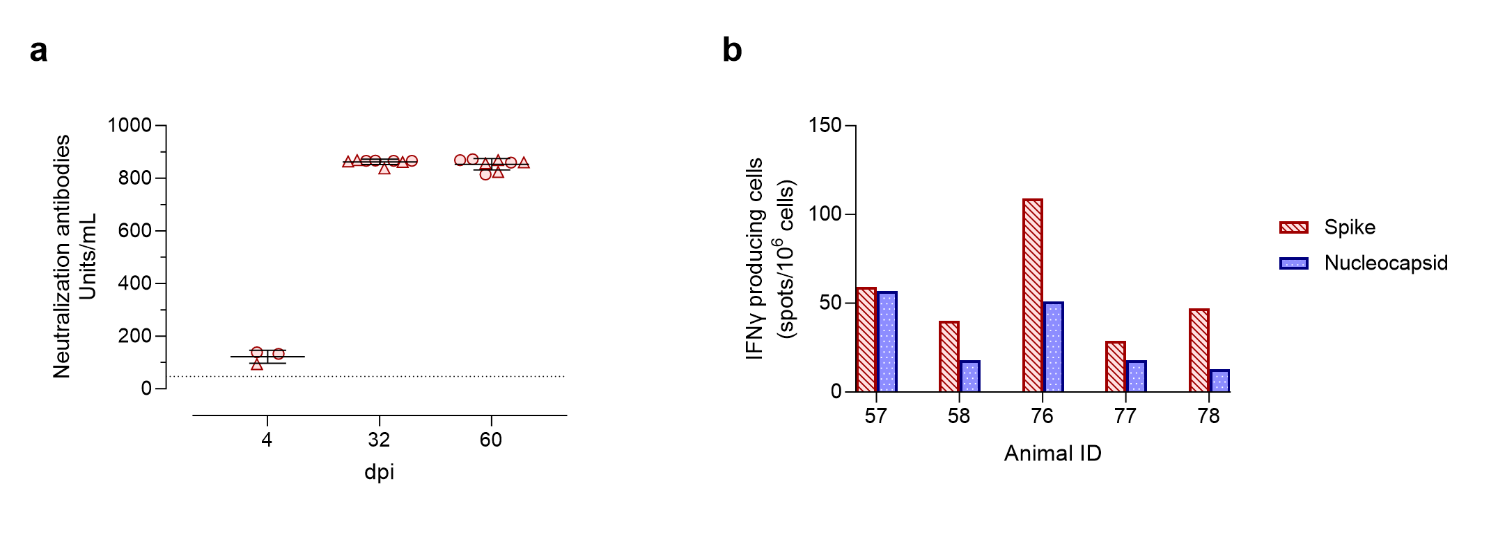
**Supplementary Figure S9.** Adaptive humoral and cellular responses to SARS-CoV-2 by inoculated hamsters. (a) Neutralizing activity (Units/mL) measured in plasma samples at 4, 32, and 60 days post-inoculation (dpi). Each point represents an individual animal; females are indicated by circles and males by triangles. The dotted line denotes the assay’s lower detection limit (47 U/mL). (b) Frequencies of IFNγ-producing splenocytes (spots per 10⁶ cells) measured by ELISpot after stimulation with SARS-CoV-2 Spike protein (red striped bars) and Nucleocapsid protein (blue dotted bars) in male (IDs 57, 58) and female (IDs 76, 77, 78) hamsters. Data show background-subtracted values (stimulated minus unstimulated wells) for the SARS-CoV-2 group only. Negative control animals exhibited no antigen-specific IFNγ response, with Spike- and Nucleocapsid-stimulated wells showing spot counts equal to or lower than unstimulated wells and are therefore excluded from the graph.


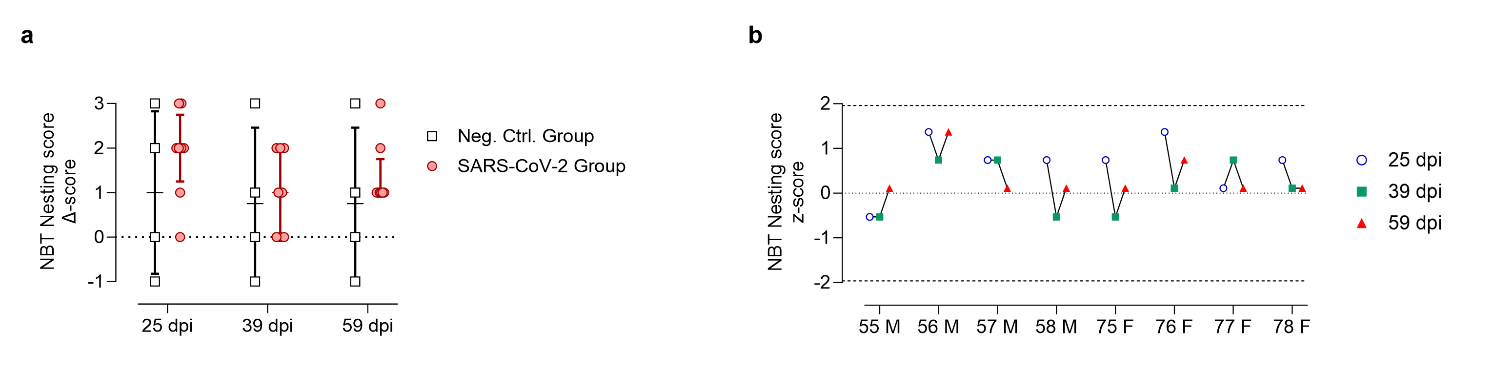


**Supplementary Figure S10.** Nest building test results at 25, 39, and 59 days post-SARS-CoV-2 inoculation. **(A)** Change in nest building test score from baseline (Delta, Δ score) in SARS-CoV-2-inoculated animals (circles, red) and negative controls (squares, grey) at 25, 39, and 59 days post-inoculation (dpi). Individual data points are shown, with group means ± standard deviation. **(B)** Individual z-scores for burrowing performance in SARS-CoV-2-inoculated males (M, IDs 55-58) and females (F, IDs 75-78) at 25 (circles, blue), 39 (squares, green), and 59 (triangles, red) dpi. Z-scores indicate the number of standard deviations each observation deviates from the control group mean. Dashed lines indicate the ± 1.96 z-score threshold for statistical significance; the dotted line represents a z-score of 0.


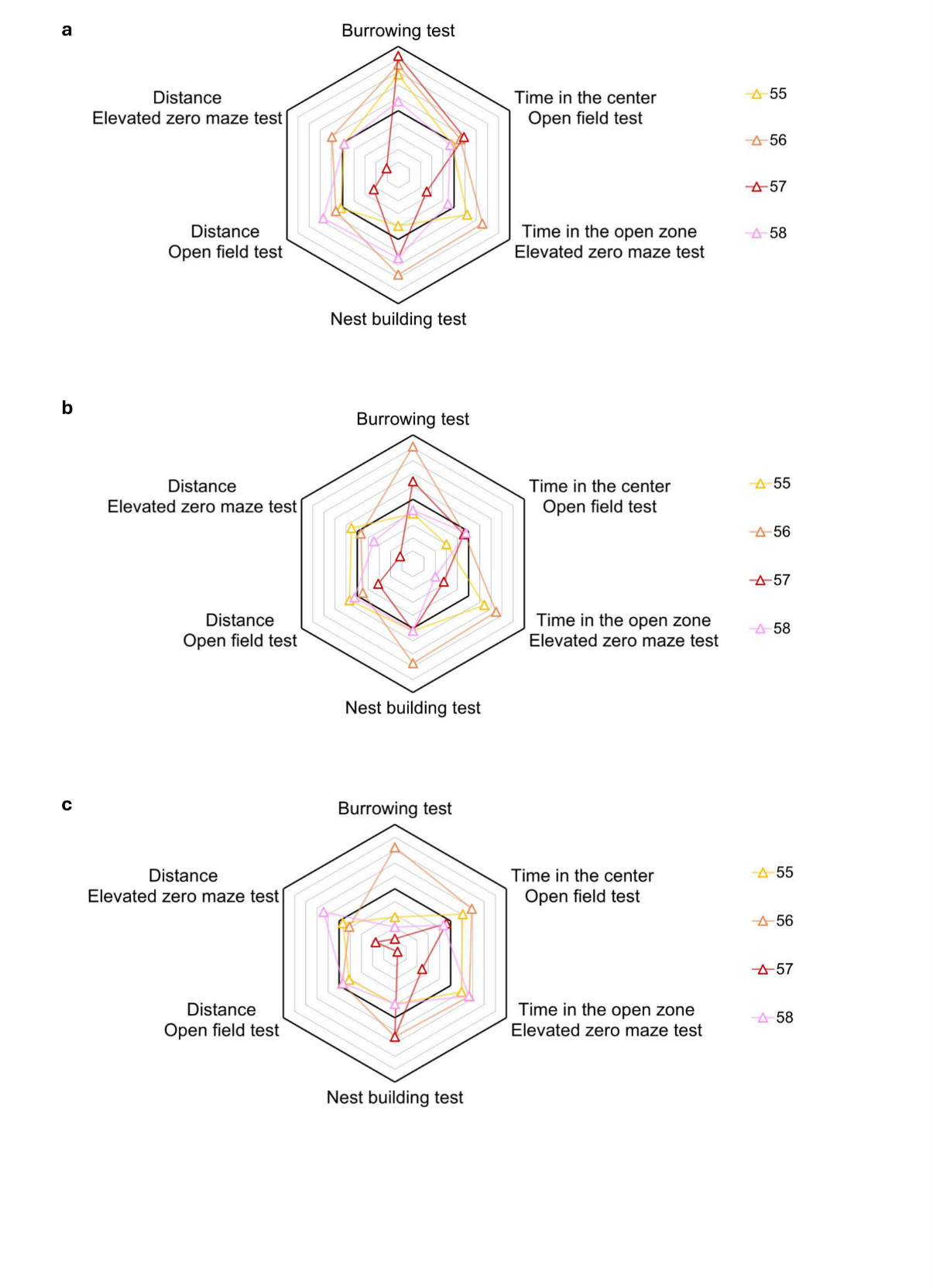


**Supplementary Figure S11.** Overall behavioral performance in male hamsters at 25, 39, and 59 days post-SARS-CoV-2 challenge, shown as z-scores derived from delta (Δ) values in the burrowing (BT), nest building (NBT), open field (OFT), and elevated zero maze (EZMT) tests. Panels depict individual z-scores for males at **(A)** 25 dpi, **(B)** 39 dpi, and **(C)** 59 dpi. Z-scores represent the number of standard deviations each Δ value deviates from the mean of the control group. The BT scores were sex-normalized and pooled across time points due to significant sex effects but no time-dependent differences (n = 6 controls per sex). The NBT, OFT, and EZMT scores were standardized using pooled controls across sex and time, as neither factor significantly affected performance (n = 12 controls). The four inner grid lines represent negative z-scores (underperformance relative to baseline and controls), the thick black line marks zero (no deviation), and the outer lines correspond to positive z-scores (increased activity).


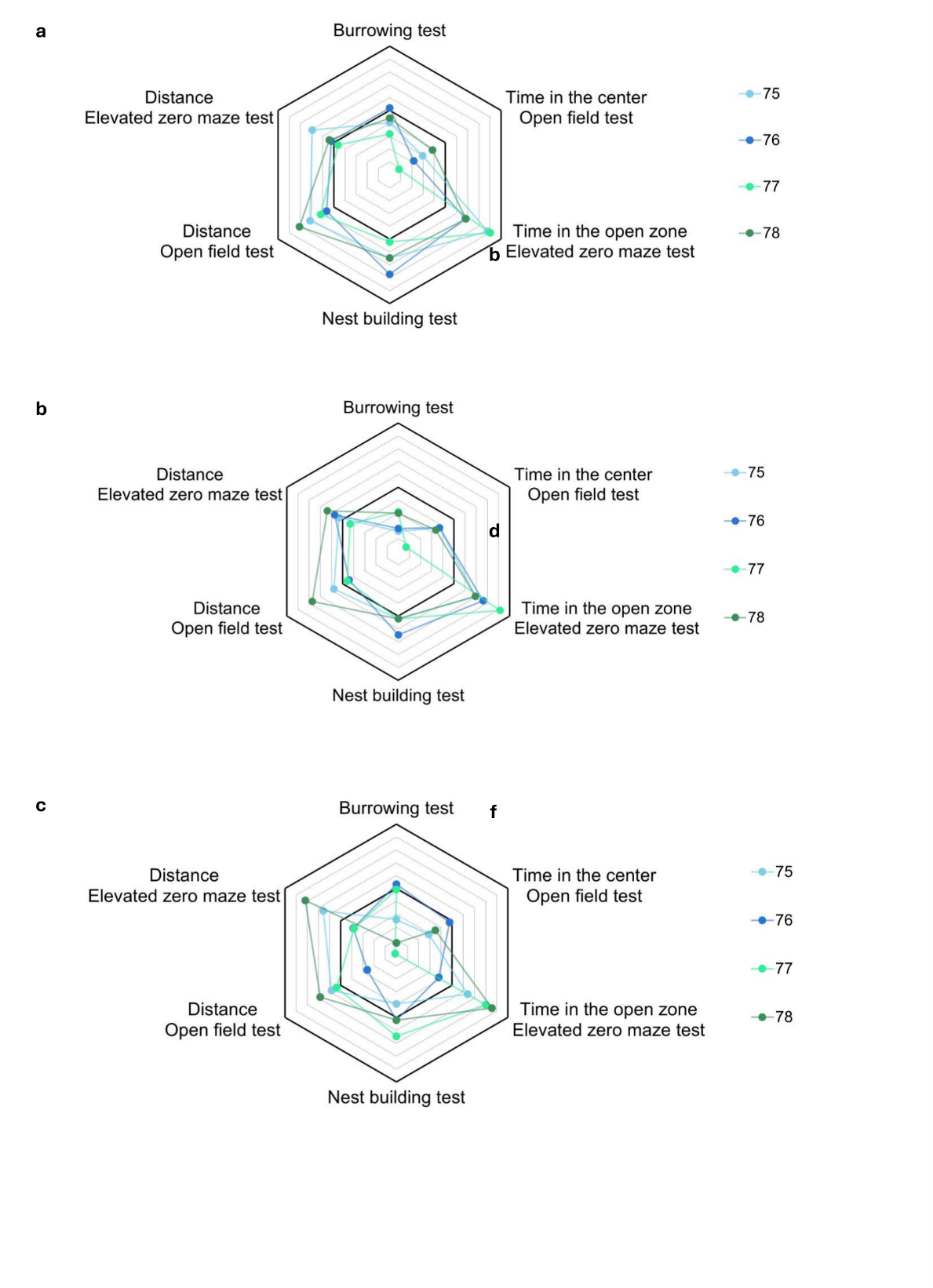


**Supplementary Figure S12.** Overall behavioral performance in female hamsters at 25, 39, and 59 days post-SARS-CoV-2 challenge, shown as z-scores derived from delta (Δ) values in the burrowing (BT), nest building (NBT), open field (OFT), and elevated zero maze (EZMT) tests. Panels depict individual z-scores for females at **(A)** 25 dpi, **(B)** 39 dpi, and **(C)** 59 dpi. Z-scores represent the number of standard deviations each Δ value deviates from the mean of the control group. The BT scores were sex-normalized and pooled across time points due to significant sex effects but no time-dependent differences (n = 6 controls per sex). The NBT, OFT, and EZMT scores were standardized using pooled controls across sex and time, as neither factor significantly affected performance (n = 12 controls). The four inner grid lines represent negative z-scores (underperformance relative to baseline and controls), the thick black line marks zero (no deviation), and the outer lines correspond to positive z-scores (increased activity).

## Supplementary Videos

**Supplementary Video S1.** Golden Syrian hamster engaged in the burrowing test.

**Supplementary Video S2.** Spontaneous behavior in the open field test.

**Supplementary Video S3**. Spontaneous behavior in the elevated zero maze test.
